# Broadening the capture of natural products mentioned in FAERS using fuzzy string-matching and a Siamese neural network

**DOI:** 10.1038/s41598-023-51004-4

**Published:** 2024-01-13

**Authors:** Israel O. Dilán-Pantojas, Tanupat Boonchalermvichien, Sanya B. Taneja, Xiaotong Li, Maryann R. Chapin, Sandra Karcher, Richard D. Boyce

**Affiliations:** 1 Department of Biomedical Informatics, University of Pittsburgh, Pittsburgh, USA; 2 Intelligent Systems Program, University of Pittsburgh, Pittsburgh, USA; 3 School of Pharmacy, University of Pittsburgh, Pittsburgh, USA

**Keywords:** Scientific data, Machine learning, Natural products, Public health

## Abstract

Increased sales of natural products (NPs) in the US and growing safety concerns highlight the need for NP pharmacovigilance. A challenge for NP pharmacovigilance is ambiguity when referring to NPs in spontaneous reporting systems. We used a combination of fuzzy string-matching and a neural network to reduce this ambiguity. Our aim is to increase the capture of reports involving NPs in the US Food and Drug Administration Adverse Event Reporting System (FAERS). For this, we utilized Gestalt pattern-matching (GPM) and Siamese neural network (SM) to identify potential mentions of NPs of interest in 389,386 FAERS reports with unmapped drug names. A team of health professionals refined the candidates identified in the previous step through manual review and annotation. After candidate adjudication, GPM identified 595 unique NP names and SM 504. There was little overlap between candidates identified by each (Non-overlapping: GPM 347, SM 248). We identified a total of 686 novel NP names from FAERS reports. Including these names in the FAERS collection yielded 3,486 additional reports mentioning NPs.

## Introduction

Recently, there has been an increase in the sales and consumption of herbal supplements for complementary health^1^. However, there are gaps in the current understanding of the safety concerns from the use of herbal or natural products (NPs), including adverse effects from the NPs and from potential NP-drug interactions that can occur due to the co-consumption of NPs and pharmaceutical drugs^2^. For example, NPs such as garlic, green tea, and ginseng can modify the effect of the prescription anticoagulant warfarin, either potentiating or reducing its efficacy leading to an increased risk of bleeding or stroke from blood clots, respectively^3–5^. By natural products, we refer to products consisting of complex chemicals produced by living organisms. Our current focus is on botanical products intended for human consumption. The constituents of these products may interact across multiple biological systems in complex ways to contribute to their effects^6^.

A promising approach to assess safety concerns for NPs is a retrospective pharmacovigilance analysis of adverse event reports from spontaneous reporting systems such, as the FDA Adverse Event Reporting System (FAERS)^7,8^. A major challenge in pharmacovigilance for NPs is the need for more standardization for coding events involving NPs. The lack of standardization in adverse event reports related to NPs leads to challenges in parsing and identifying the products' names and ingredients due to their non-uniform representation in the reports^8,9^. Therefore, researchers often encounter unfamiliar NP names or spelling variations when identifying reports for pharmacovigilance^2^. For example, the FAERS database includes more than forty-four (44) names referring to "Licorice" including "Liquorice", "*Glycyrrhiza glabra*", and "*Glycyrrhiza laevis.*"

Equation (1) Gestalt Pattern-Matching ($GPM(X,Y)$)

${s}_{1}:$ Longest Common Substring between $X \& Y$

${s}_{n}:$ Subsequent Common Substring between $X \& Y$

1 $$GPM\left( {X,Y} \right) = { }\frac{{2 \cdot \left( {\left| {s_{1} } \right| + \ldots + \left| {s_{n} } \right|} \right) }}{\left| X \right| + \left| Y \right|}$$

Equation (2) Normalized Levenshtein Distance ($Lev(X,Y)$)

2 $$lev\left( {X,Y} \right) = \left\{ {\begin{array}{*{20}l} {\left| X \right|} \hfill & {if\left| Y \right| = 0} \hfill \\ {\left| Y \right|} \hfill & {if\left| X \right| = 0} \hfill \\ {lev\left( {tail\left( X \right),tail\left( Y \right)} \right)} \hfill & {ifX_{0} = Y_{0} } \hfill \\ {1 + {\text{min}}\left\{ {\begin{array}{*{20}l} {lev\left( {tail\left( X \right),Y} \right)} \\ {lev\left( {X,tail\left( Y \right)} \right)} \\ {lev\left( {tail(X} \right),tail(Y))} \\ \end{array} } \right.} \hfill & {otherwise,} \hfill \\ \end{array} } \right.$$

$$Lev\left(X, Y\right)=\frac{lev\left(X,Y\right)}{{\text{max}}\left(\left|X\right|, \left|Y\right|\right)}$$

Previous work has used fuzzy string-matching to overcome this limitation^10^. This approach helps mitigate the effects of similar name variations and misspellings but does not fully bridge the gap between the spectrum of names referring to the same product^8,10^; such as matching the common name “Liquorice” to its equivalent Latin binomial name “*Glycyrrhiza glabra*”.

Equation (3) Cosine Distance ($CD\left(X,Y\right)$)

3 $$CD\left(X,Y\right)=1-\frac{{\sum }_{i=1}^{n}{X}_{i}{Y}_{i}}{\sqrt{{\sum }_{i=1}^{n}{X}_{i}^{2\sqrt{{\sum }_{i=1}^{n}{Y}_{i}^{2}}}}}$$

**Figure 1:**
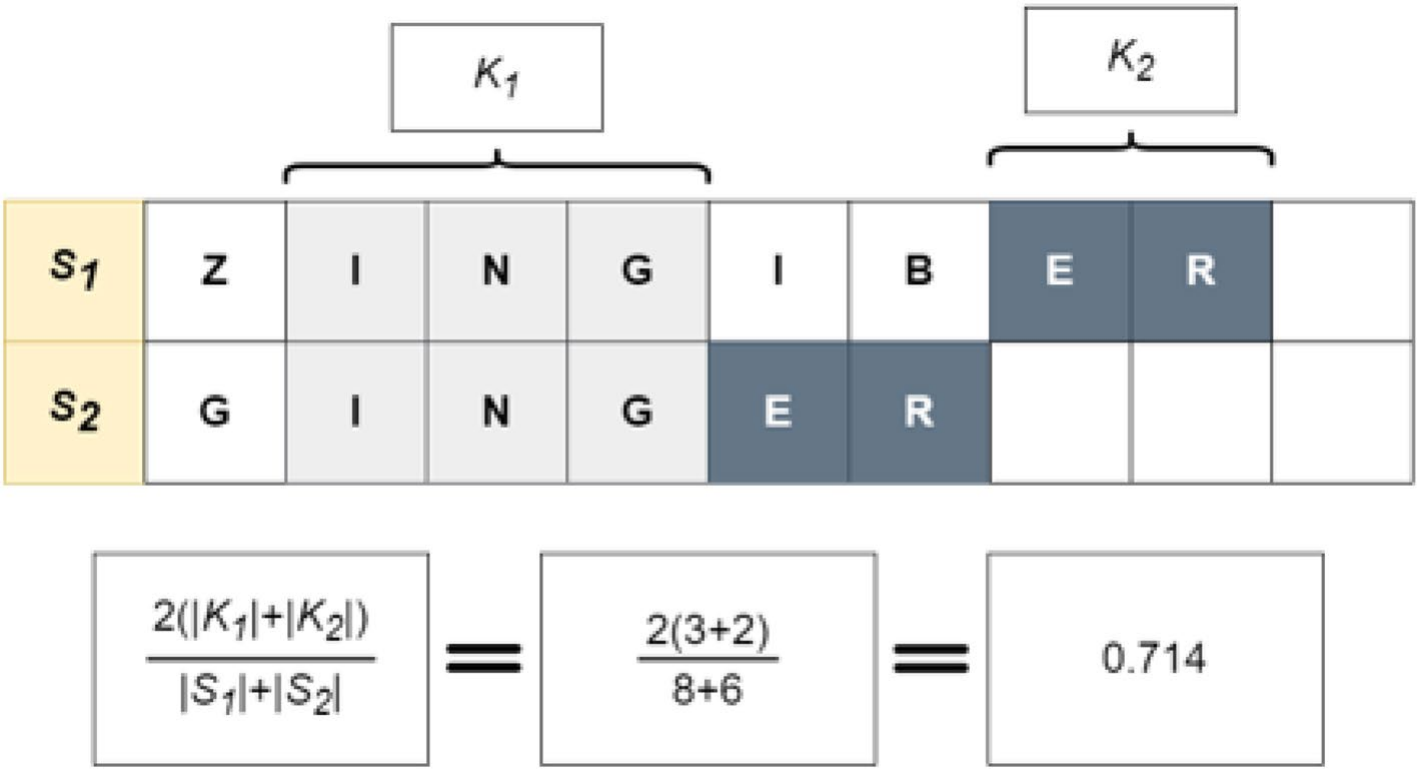
Example of Fuzzy String-Matching (GPM): Finding the similarity between the terms S_1_: “ZINGIBER” and S_2_: “GINGER” using the Gestalt Pattern-Matching approach. The longest matching substring K_1_: “ING” servers as an anchor to align the inputs. Next, the recursion matching happens, matching substring to the left and right of K_1_, here K_2_: “ER” represents a second matching substring of characters to the right of K_1_. The calculation of a similarity score based on GPM is shown at the bottom of the figure.

**Figure 2:**
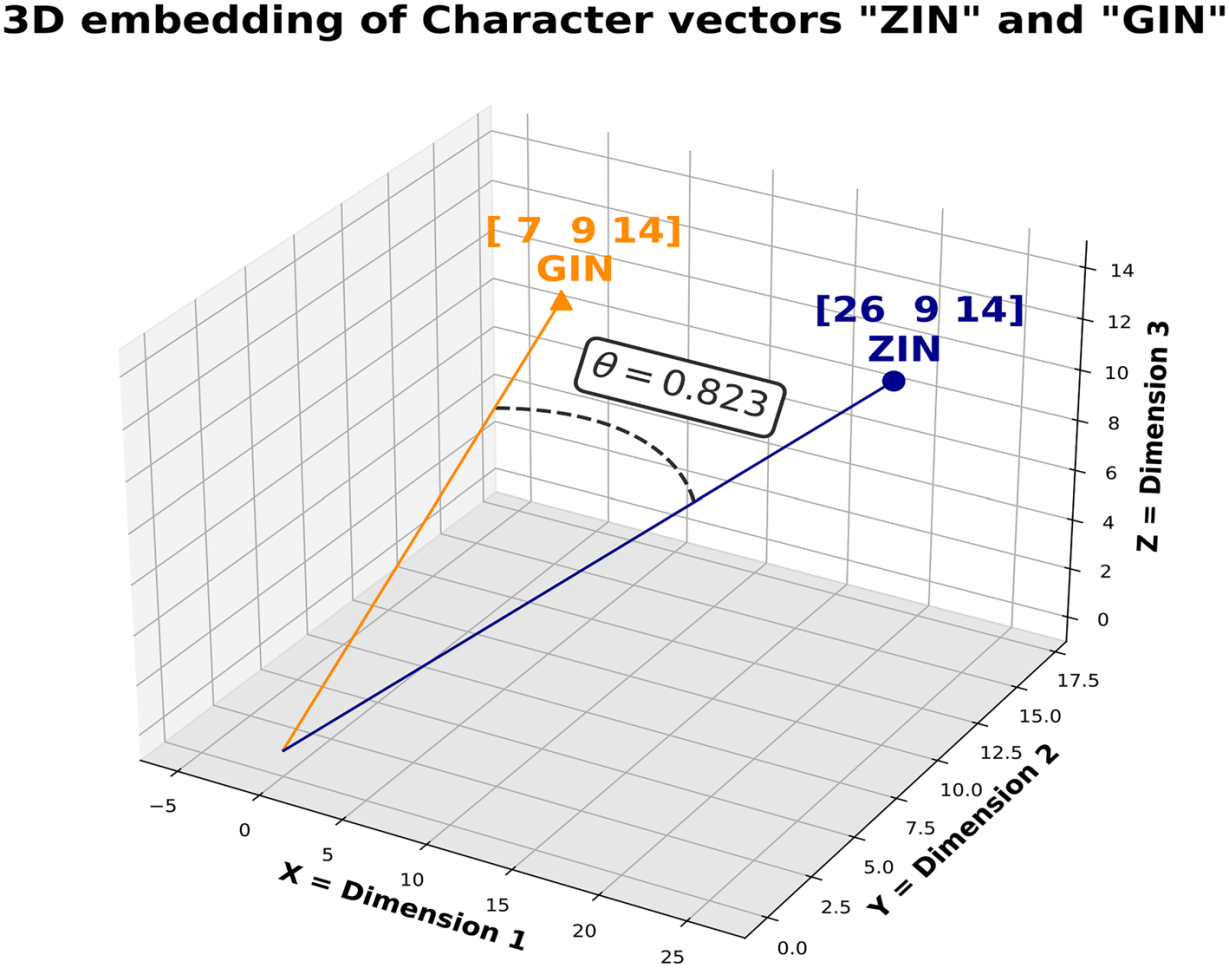
Example of Cosine Distance: This example shows the Cosine Similarity Θ between GIN and ZIN. The length of the string in the example has been reduced to three characters to allow a 3-dimentional representation. The Cosine Distance is calculated as 1 – Θ.

**Figure 3:**
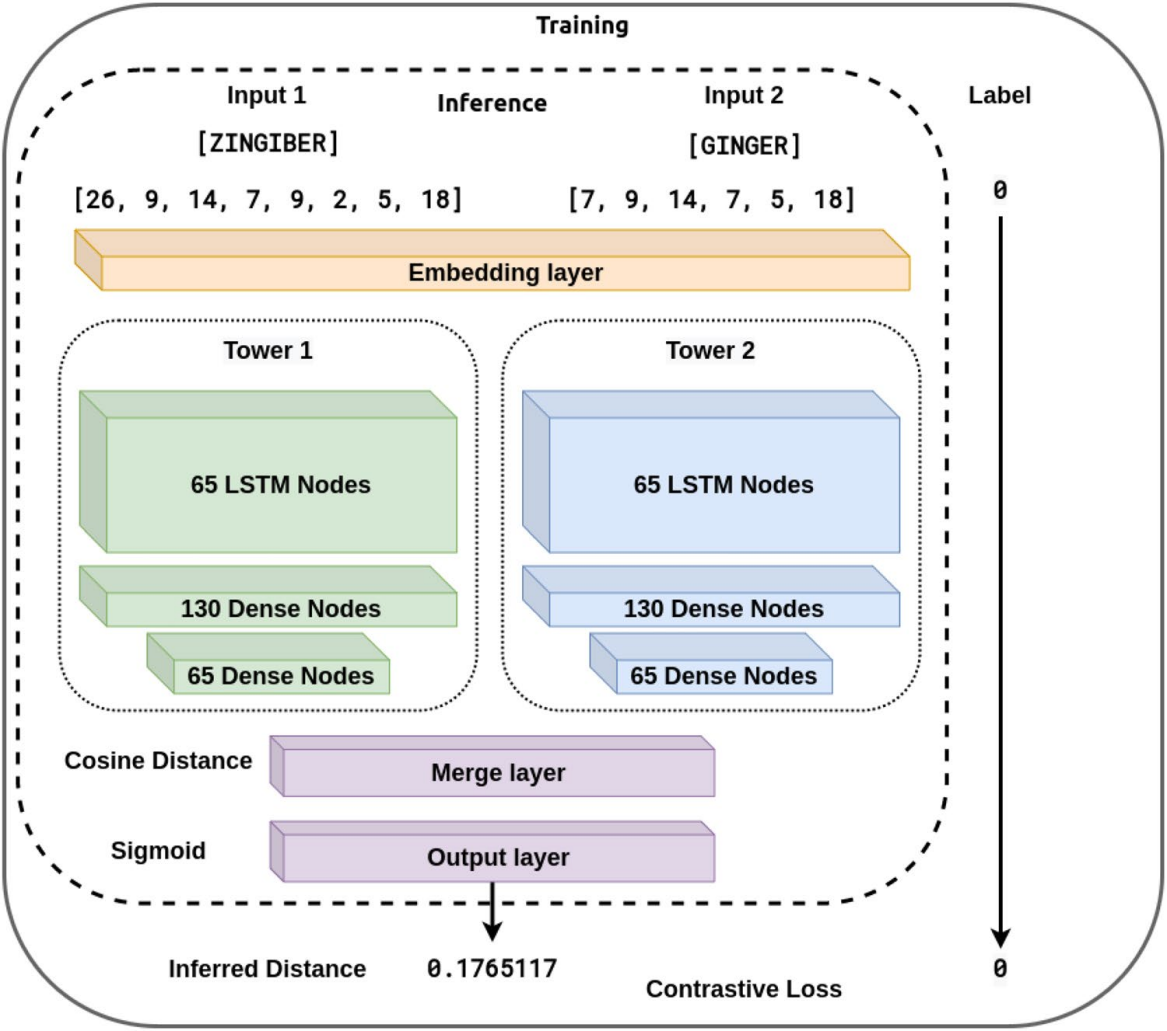
Model Architecture: This Siamese model architecture diagram shows the inputs for the training forward passes within the solid box and the input for the inference forward passes inside the dotted box. Read from top to bottom, during the inference forward passes, the inputs are first mapped to integer values, then passed into the Embedding Layer to produce the embeddings, which serve as the input to the two Siamese towers, whose output is combined in the Merge layer using the Cosine Distance, whose value gets passed to the output layer producing an inferred distance between the input terms. Additionally, the solid box shows the label that would be used in the supervised training step to calculate the contrastive loss and backpropagation.

To address these shortcomings, we propose combining fuzzy string-matching and deep learning to broaden the capture of candidate NP names. A combination approach can leverage both the reliability of fuzzy string-matching and the flexibility of deep learning to identify both spelling variations and alternative names for a given product name. For example, given a misspelled form of Licorice, such as "Likorice", the model will be able to map it to its Latin binomial name, "*Glycyrrhiza glabra*" and its other species by outputting a small distance between them. For this work, we utilized Gestalt pattern-matching^11^ (GPM) as the fuzzy string-matching component to maximize the identification of candidate spelling variations (Eq. 1 and Fig. 1). The proposed deep learning approach relies on the cosine distance (Eq. 3 and Fig. 2) between learned embeddings to create a model that matches NP names. The deep learning approach is based on the Siamese model (SM) architecture (Fig. 3). The SM architecture facilitates learning the embeddings by comparison of the inputs through the contrastive loss function (Eq. 4). The Siamese neural network was chosen for this task because they have been shown to successfully address the challenge of identifying similarities over a considerable range of problems^12^. Given an unknown term and a set of alternatives, the model learns to embed the inputs to minimize the cosine distance between terms that are spelled similarly or that are semantically similar. Additionally, they have been successfully trained with relatively little data^13^.

Equation (4) Contrastive Loss ($CL\left(X,Y\right)$)

4 $$CL\left({x}_{i},{x}_{j},\theta \right)=1\left[{y}_{i}={y}_{j}\right]{\left|\left|{f}_{\theta }\left({x}_{i}\right)-{f}_{\theta }\left({x}_{j}\right)\right|\right|}_{2}^{2}+1\left[{y}_{i}\ne {y}_{j}\right]{{\text{max}}\left(0, \varepsilon -{\left|\left|{f}_{\theta }\left({x}_{i}\right)-{f}_{\theta }\left({x}_{j}\right)\right|\right|}_{2}\right)}^{2}$$

We also explored Levenshtein Edit Distance (LED) as another form of fuzzy string-matching. LED’s algorithm (shown in Eq. 2) presents a way to quantify the number of edits necessary to transform a query sequence into a target sequence by recursively comparing the characters in each position of the sequence. We opted not to include LED in the experiment seeking novel spelling variations for the following reasons. First, LED is a default fuzzy string-matching algorithm in many query systems, meaning the variations it could identify might already be present in the query set. Second, the results from the comparison experiment indicated that LED was comparable to GPM. And third, including LED in the novelty experiment would increase the burden on the team performing the manual validation with terms that we would expect to have a high overlap with the results from GPM.

## Methods

### Data collection

The first data source was the Center for Excellence for Natural Product-Drug Interaction Research (NaPDI) Database, from which we collected the known product names of several NPs, some of the previously identified spelling variations, and their corresponding Latin binomial names^14^. A second data source was the FAERS database, from which we identified additional product names or spelling variations using fuzzy string-matching for 70 different NPs^7^. FAERS data from Q1 2004—Q2 2021 was loaded into a standardized database and manual annotation was used to map 5,358 drug name strings from adverse event reports that matched to NP names. The remaining 389,386 unmapped drug names from FAERS were used for the novelty experiment in this study.

### Experiments

The data was used to train and evaluate the Siamese model (SM) by conducting several experiments to study the effectiveness of the SM at matching potentially relevant terms from the reports to the corresponding NP names. We initially explored the SM’s performance as a distance metric to relate NP names effectively. Then, we evaluated how the SM compared to fuzzy string-matching approaches in tackling the same problem, validating that the SM can match novel names or spelling variations from FAERS to the correct equivalent group of NPs. Finally, we combined both approaches to produce a set of candidate NP names to be manually validated and utilized during FAERS report collection.

### Data pre-processing & inclusion criteria

The training data consisted of pairs of spelling variations of the product names from the manual annotation and a distance label where "1" indicated distant terms and "0" indicated matching terms. An example row of a positive matching pair might be ("Likorice", "Liquorice", 0) and a negative matching pair ("Cinnamon", "Liquorice", 1). This representation allows the Siamese Model to learn the associations between query and target terms and represent the associations as a distance between 0 and 1. For simplicity, we decided to reduce the variation across terms. To this end, the data was standardized such that any non-alphabetical characters were removed from the terms, with the only exception being the whitespace character. All characters in the terms were then capitalized. Due to limitations of the implementation of Keras’ Embedding Layer^15^, a fixed-sized cutoff for the maximum length of the terms is required, and inputs must be represented as positive integers. We chose our cutoff by choosing a number close to the sum of the average size of the terms in the data (30) plus one standard deviation (31). Therefore, terms longer than sixty-five (65) characters were discarded. The last step in this initial processing was to encode the terms into integer sequences, where each letter was mapped to its corresponding position in the English alphabet, so [A-Z] became [1–26], and the space character was mapped to the integer 27. For sequences smaller than the sixty-five (65) maximum size cutoff, 0-padding was used to pad the rest of the sequence up to the sixty-five (65) elements.

Through exploratory data analysis, we identified two sources of imbalance in our data. We found some target labels were disproportionally represented in the data and discovered that there was an additional imbalance in the proportion of matching to non-matching sequences. After this initial data processing was done, two data balancing steps were performed to reduce label imbalance. First, we balanced the representation of each target name to approximately the same amount since no target label should be overrepresented in the dataset. The additional pairs were generated by using any names matching the target name; the names were modified by adding random modifications to the query term to create new unique pairs. These random modifications were performed by first randomly selecting 40% of the characters in the query sequence, then for each of these characters a random sample was drawn from a standard uniform (0,1) distribution, the random sample determined the modification to be performed. If the sample was in the interval [0.0, 0.2), the character in that position was replaced with a new random character [A-Z] or space, if the sample was in the interval [0.2, 0.4), the character in that position was removed, if the sample was in the interval [0.4, 0.6), one random character or space was added after that position, if the sample was in the interval [0.6, 0.8), the character was transposed with the one in the previous position, and finally, if the sample was in the interval [0.8, 1.0], no modification was  performed to that position. The second balancing step was similar, in that it generated matching and non-matching pairs as necessary to balance the total number of matching and non-matching pairs in the complete dataset. After the balancing procedures were completed, the 70/30 train-validation split, and a separate test/holdout set were created. A description of the number of samples in each of the sets is provided in Table 1.

**Table 1:** Train-validation data summary.

	Matching*	Non-Matching*	Total
Train	841,259 (0.503%)	830,348 (0.497%)	1,671,607
Validation	360,717 (0.504%)	355,687 (0.496%)	716,404
Test/Holdout	1,261 (0.504%)	1,239 (0.496%)	2,500

*In the columns for Matching and Non-Matching, the first number represents the actual count number, and the second number represents what percentage of set that first number accounts for.

### Siamese model training

We utilized the SM architecture, as shown in Fig. 1. A SM comprises two identical neural network towers with the same architecture. In our implementation, each tower is made from 65 recurrent bidirectional Long Short-Term Memory (LSTM) cells^16^. The outputs of the towers were combined using the cosine distance between the vectors of the embedded terms. The contrastive loss function was utilized during training to measure the model's accuracy. The corresponding input to each tower was first embedded into a 30-dimensional space by an embedding network comprised of two (2) layers of hundred-and-thirty (130) dense nodes each. The hyperparameters for the number of dense nodes, embedding dimensions, and the number of layers in the embedding network were chosen experimentally.

### Comparison with fuzzy string-matching

To evaluate the model’s usefulness in identifying the correct matching NP name, we compared the model’s performance against a fuzzy string-matching approach. The algorithms utilized for fuzzy string-matching were the Levenshtein edit-distance (LED) (Eq. 2) as implemented in TensorFlow’s “edit_distance” and Gestalt pattern-matching (GPM) as implemented in Python’s “difflib” library “get_close_matches” function^11,17^. The LED is a metric used for comparing the similarity between two sequences based on their “edit distance.” Gestalt pattern-matching is an algorithm also used to compare the similarity between two sequences. The metric used for comparison was Mean Reciprocal Rank (MRR)^18^ (Eq. 5), with which we measured the top twenty (20) results predicted to be the most similar to the target value annotated in the dataset. Additionally, we also compared the top results to any of the product names equivalent to the target. These top twenty (20) results are used as candidate NP names to be validated further.

Equation (5) Mean Reciprocal Rank (MRR)

5 $$MRR=\frac{1}{n}{\sum }_{i=1}^{n}\frac{1}{Ran{k}_{i}}$$

### Novelty experiment

Finally, to evaluate the applicability of our methods for pharmacovigilance research, we extracted 389,385 drug name strings from the FAERS database that were not mapped to any drugs or NP names and might contain NPs. After processing the unmapped names, 7,751 were removed because they were longer than sixty-five (65) characters. Another 41,849 sequences were identified as duplicates and were also removed; the remaining 339,785 were utilized for this novelty experiment to identify unique NP names from unmapped reports. For this experiment, we utilized a subset of 70 NPs of interest (the 70 natural products chosen were mentioned by a 2020 Market Report^19^ and/or were of interest to the NaPDI Center) from the set of NP names used for training. This subset contains both the Latin Binomial and a known common name, referred to as the preferred term (PT) for each of the seventy (70) natural product name pairs. These hundred-and-forty (140) names were utilized as a query set to identify candidate mappings from terms found in the FAERS database. We then utilized GPM and SM to match the top twenty (20) unmapped FAERS strings with results predicted as the most similar to (least distant to) the query terms. In this experiment, we explore the combined results of GPM and the SM, LED was not included.

### Manual validation

The candidate mappings between the query NP names and unmapped FAERS strings yielded by the novelty experiment were manually annotated by two health professionals to assess whether the candidate mappings were correct. This process aims to leverage their expertise with drug and NP names to validate the results from the model. We further corroborated the annotations through Cohen's kappa interrater agreement metric (Eq. 6) and an adjudication process to resolve the points of disagreement^20^.

Equation (6) Cohen's Kappa Interrater Agreement

${p}_{0}:$ Relative observed agreement among raters.

${p}_{e}:$ Hypothetical probability of chance agreement

6 $$k=\frac{\left({p}_{0}-{p}_{e}\right)}{\left(1-{p}_{e}\right)}$$

## Results

### Model training results

**Figure 4:**
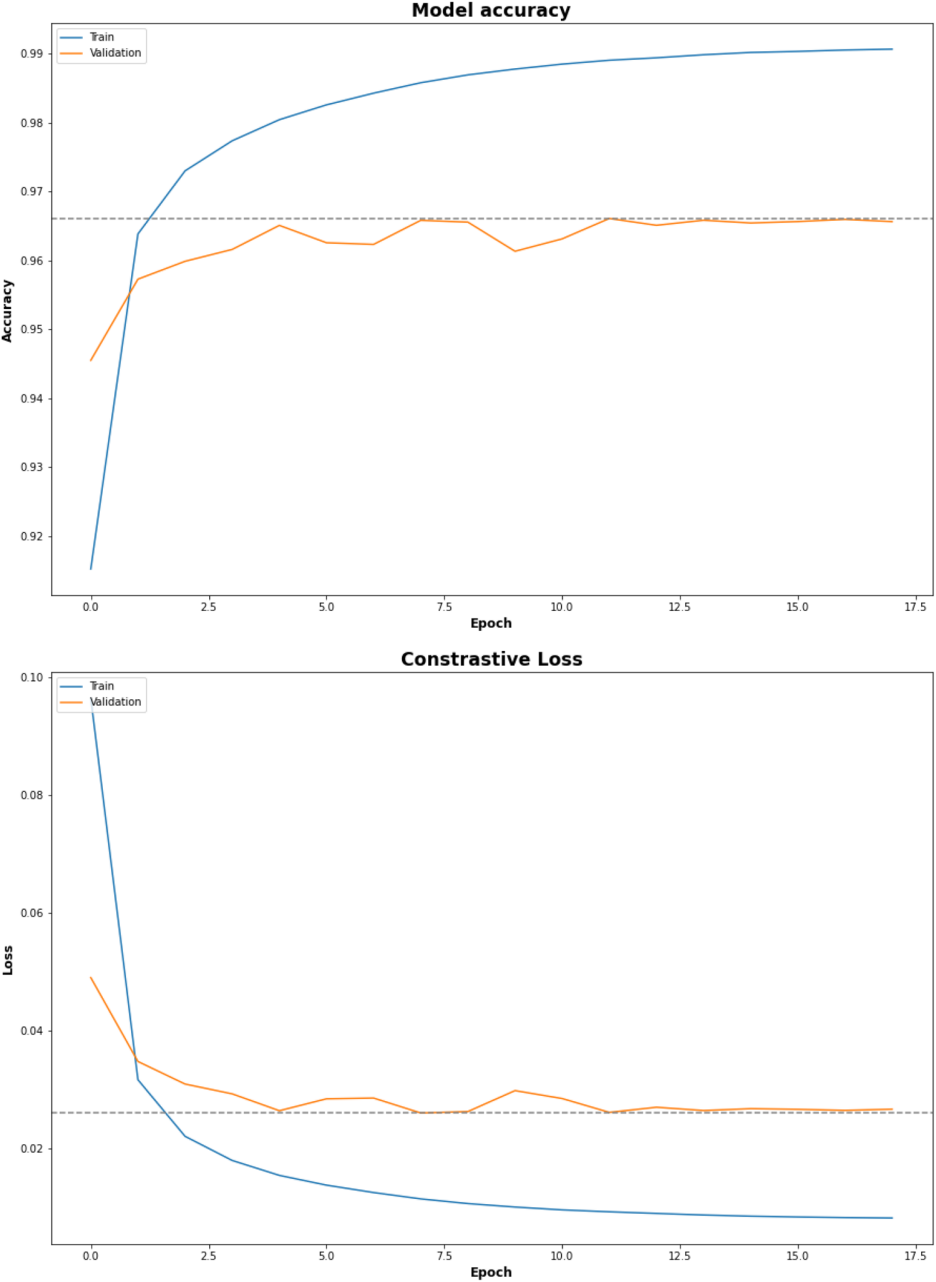
Siamese Model Training Results: The accuracy and loss of the Siamese model during training and evaluation both followed a similar trend, reaching a maximum of 97% accuracy with the validation set. The blue line represents the performance of the model on the training set and the orange line represents the performance of the model on the validation set. The dotted line represents the maximum or minimum value in the graph.

After training the SM for up to five hundred (500) epochs, the model terminated early at seventeen (17) epochs (Fig. 4). The best-performing epoch in this training run achieved a validation accuracy of 0.97 (validation loss: 0.03). The weights from that epoch were saved and utilized for the rest of the experiments.

A holdout set containing 2500 pairs was utilized to compare the MRR performance. For the MRR evaluation, we were only interested in a subset of the matching pairs (n = 1,000) given that we used the first element of the pair as the query and the second element as an indicator of the correct answer. Using the top 20 NP names reported as the least distance to the query term by each approach, we looked for exact matches to the target pair and matches to terms equivalent to the target pair.

**Figure 5:**
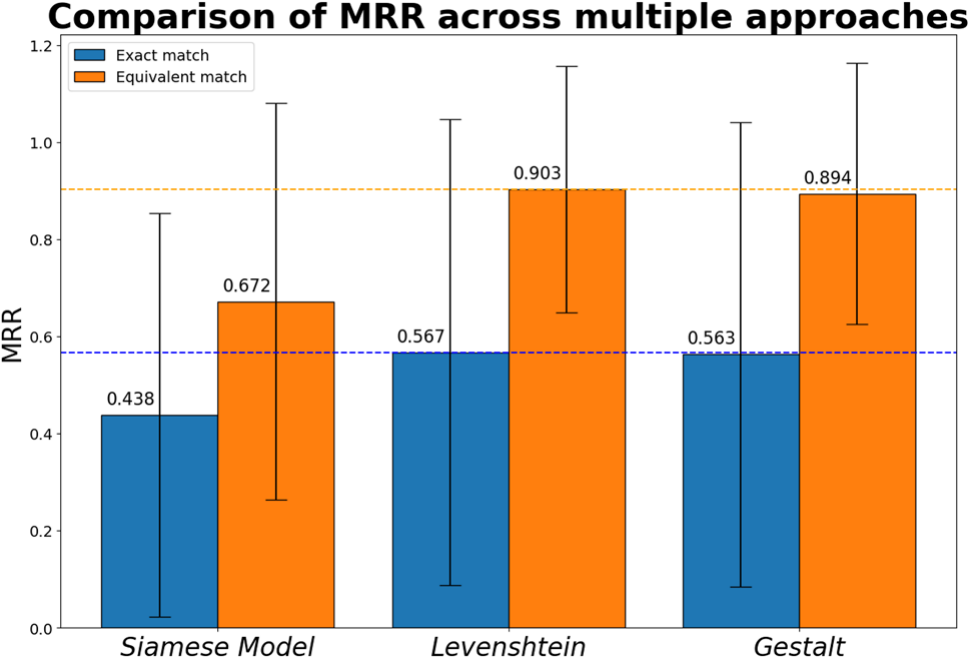
Comparison between Siamese Model and Fuzzy String-Matching: The Gestalt Pattern-Matching (GPM) approach performed best with a higher Mean Reciprocal Rank score in the exact matching of terms, whereas the Levenshtein Edit-Distance (LED) approach performed better in the matching of equivalent terms. The blue dotted line represents the maximum exact MRR across the methods compared (LED, 0.567) and the orange dotted line represents the maximum equivalent MRR (LED, 0.903) across the methods.

For the exact matching where $X\sim Y$, the LED approach performed best (MRR = 0.567). In the equivalent matching where $X\sim Y^{\prime } |Y \in Y^{\prime }$, the LED approach also performed best with (MRR = 0.903). In both cases, the GPM approach performed similarly to LED with slightly lower MRR scores (exact = 0.563, equivalent = 0.894.) In both tests, the SM achieved comparably lower MRR scores (exact = 0.438, equivalent = 0.672.) see Fig. 5 and Table 2.

**Table 2:** Results from mean reciprocal rank comparison.

	Exact	Equivalent
Siamese model	0.438 (SD 0.416)	0.672 (SD 0.408)
Levenshtein distance	0.567 (SD 0.480)	0.903 (SD 0.254)
Gestalt pattern-matching	0.563 (SD 0.478)	0.894 (SD 0.269)

### Novelty results

The single-blind test evaluation showed strong agreement (Kappa = 0.86) between the annotators on the identified candidate mappings. The specificity of the identified terms was the primary cause of disagreements between the annotators. In the presence of disagreements, the rules in Table 3 were utilized for adjudication. After adjudication, evaluators reported that the SM identified 504 correct terms, and GPM identified 595 (Table 4). For the 70 NPs of interest, we considered those where one or more correctly identified NPs were covered by the approach (Table 5). When comparing these results, the GPM and SM approaches performed similarly, respectively identifying an average of 6 and 5 reports for the products they covered. From this novelty experiment, we were able to identify a total of 158 novel NP names and spelling variations for 70 NPs.

**Table 3:** Adjudication rules.

Rules	
If the query term was a Latin Binomial and the result was a general common name or only a Genus name, this was identified as a non-match.	
​If the query term was a Common name and the result could potentially collide with another medical term, this was identified as a non-match.

**Table 4:** Total products identified through each approach (including duplicates).

	GPM	SM	Combined
Latin binomials	278	221	499
Common Names	317	283	600
Total	595	504	1,099

**Table 5:** Coverage of identified products of interest.

	Latin binomial	Common name	Total
Gestalt pattern-matching	44	53	97
Siamese model	45	49	94

### Manual validation

It is worth noting that many of the terms did not overlap between the approaches (Table 6). The SM identified 248 unique names, while GPM identified 347. The unique terms obtained from this mapping were incorporated into our quarterly data collection from FAERS data between Q1 2004 and Q2 2022^21^. For mining reports containing mentions of NPs, we only looked at the reports involving the products for which the novel product names were identified; these 57 NPs are a subset of the original 70 NPs of interest. Including the novel terms from the experiments above resulted in the capture of 3,486 additional reports that were not previously identified in the database (Table 7).

**Table 6:** Unique products identified by each approach (excluding overlap).

	GPM	SM	Combined
Latin binomials	163	107	270
Common names	184	141	325
Total	347	248	**595**

Significant values are in bold.

**Table 7:** FAERS reports collected before and after the inclusion of novel names.

	Before	After	Difference
Reports	48,694	52,180	3,486

## Discussion

This study combined fuzzy string-matching and Siamese neural network approaches to identify NP names in adverse event reports in the FAERS database and successfully broadened the capture of NP reports by approximately 7.5%. Prior work in using string matching methods to identify NP strings in spontaneous reporting systems have used multiple sources of NP names to create a thesaurus to identify adverse event reports^8,20^. This requires maintenance of the thesaurus and regular updates to capture relevant NPs and name variations. This study expands upon the prior work that uses string matching using a manually annotated dataset from the FAERS database that can be used to train the model to identify NP variations. The approach can also be effectively utilized to broaden the capture of reports in other spontaneous reporting systems and overcome challenges in NP pharmacovigilance, including lack of interoperability among NP data sources, lack of coverage of synonyms, scientific names and common names, and ambiguity in NP names in adverse event reports^8^. The manual annotation results showed that both approaches contribute sufficient unique candidate mappings that help increase the number of reports identified in FAERS, which is essential considering that only 0.4% of the reports in FAERS involve NPs. Using a combination of fuzzy string-matching and a Siamese Neural Network, we increased our capture of relevant reports by approximately 7.5%.

### Combined approach

We trained a SM to serve as a proxy distance metric for identifying potential spelling variations of NP names. Looking at the results from the training process, it is encouraging to see the potential of the method in tackling the problem of mining emerging variations in adverse event reports. In agreement to previous work that suggests natural language processing approaches can outperform current methods^8^, we expected SM to outperform fuzzy string-matching approaches. During our work, it was clear that this was not the case with our current implementation. Although the approach minimized the distance between similar terms, as seen during the training evaluation, it did not effectively maximize the distance between dissimilar ones, as suggested by the MRR comparison. This may be due to potential overlaps between spelling and semantical similarities of the query and target space.

Potential limitations with the training of the SM includes the completeness of the data, shortcomings of the evaluation metrics, and the generalizability of the methods. Due to the nature of the problem, the data on spelling variations for NPs utilized for training was in no way complete or exhaustive. Our approach to data processing and augmentation lends itself to increasing the model's capacity to generalize novel variations at the risk of saturating and confounding the embedding space. As implemented, the SM is learning two different tasks, one for "denoising" the spelling variations to the preferred term and another for matching equivalent terms as similar. Separating these tasks and creating a model architecture for the specialized handling of each task might prove advantageous. In the current work, the MRR metric only measures the top response and not the results' completeness. Tweaking this aspect of how we measured MRR might provide a more accurate assessment of the applicability of the approaches.

We chose the SM architecture for this work because it possesses the following qualities. It can easily be used for distance metric learning between pairs. Siamese models have been shown to successfully learn distance metrics even with little data. The SM approach was, at most, only comparable to approaches such as LED and GPM. Nonetheless, such an approach proved helpful in mining adverse event reports for mentions of NPs, as seen in the novelty experiment. The novel NP names identified in the novelty test (supplementary material) will help refine the task of mining natural products from adverse event reports (AERs) in the future.

### Limitations

We encountered some limitations in our implementation, such as the need for a fixed input size. Since the average length of the name of the NPs considered for the study was thirty (30) characters with a standard deviation of thirty-one (31), we chose a value close to the mean plus the standard deviation for our sequence length cutoff. In turn, the current model targets sequences of up to sixty-five (65) characters, approaches that might enable us to generalize applicability past this threshold are desirable. This means that currently, we cannot process sequences longer than sixty-five (65) characters. A second limitation was identified in the MRR comparison experiment. For the current problem, the orthographical and semantical spaces are not mutually exclusive; overlaps between spelling similarity and semantical dissimilarity and vice-versa can hurt the model's performance. Another limitation of our work is that candidate names were mined for only 70 NPs of interest. Another area for improvement is that, as implemented, our model did not prioritize semantic similarity over spelling similarity, leading to increased misidentified candidate NP names. Finally, the scalability of the manual validation process presents a hurdle as the amount of candidate names increases.

### Future work

Our future work will involve assessing how different elements, such as the amount of noise used in data processing and the size of the train/validation data split, impact the model's training performance. We also plan to investigate alternative ways of handling data processing, including adding features to the data and creating model architectures that separately consider orthographical and semantic similarity. Moreover, we aim to expand our candidate identification process by mining candidates for a broader range of natural products. We will prioritize semantic similarity over spelling similarity to improve accuracy. Additionally, we will focus on enhancing the reliability of our methods to reduce the need for manual validation. We believe it is important to continue this work. As our methods of identifying the mention of NPs in AERs improve, we expect to pick up more NaPDI signals, enhancing patient safety through NPs pharmacovigilance.

## Conclusion

A SM was trained to identify potential spelling variations of NP names. The SM model training terminated early at seventeen (17) epochs, achieving a validation accuracy of 0.97. In MRR evaluation, the SM performance was, at most, comparable to that of the fuzzy string-matching approaches. In the novelty experiment, GPM and SM performed similarly in identifying correct terms. The unique terms obtained were incorporated into the quarterly data collection process, resulting in the capture of 3,486 additional reports. By combining both the SM and GPM, a broader capture of NP names was achieved. Nonetheless, careful manual validation is still required for validation of the identified candidate names. Through this process of novel NP name discovery and interaction detection, we can help further research on natural product drug interactions.

### Supplementary Information

Supplementary Information.

## Data Availability

The full list of natural product names identified in FAERS for the 70 NPs of interest can be found in the supplementary material. The data utilized for both training the Siamese Model and the identification of NP candidates through the combined approach is available as open access data through Zenodo: https://doi.org/10.5281/zenodo.8155759.

## Code availability

The code and data utilized for this work are available from the following GitHub: https://github.com/dbmi-pitt/np_name_finder. The repository includes the code, configuration files, and data required to reproduce the work.

## References

[1] Smith, T., Resetar, H. & Morton, C. US Sales of Herbal Supplements Increase by 9.7% in 2021. *J. Am. Botanical Council* **19**,.

[2] Sharma V, Sarkar IN (2017). Identifying natural health product and dietary supplement information within adverse event reporting systems. Biocomputing.

[3] Leite PM, Martins MAP, de GraçasCarvalho M, Castilho RO (2021). Mechanisms and interactions in concomitant use of herbs and warfarin therapy: An updated review. Biomed. Pharmacotherapy.

[4] Gouws C, Hamman JH (2020). What are the dangers of drug interactions with herbal medicines?. Expert Opinion Drug Metabolism Toxicol..

[5] Tan CSS, Lee SWH (2021). Warfarin and food, herbal or dietary supplement interactions: A systematic review. Br. J. Clin. Pharmacol..

[6] Natural Products Research—Information for Researchers. *NCCIH* https://www.nccih.nih.gov/grants/natural-products-research-information-for-researchers.

[7] Research, C. FDA Adverse Event Reporting System (FAERS) Public Dashboard. *FDA* (2019).

[8] Sharma V, Gelin LFF, Sarkar IN (2020). Identifying Herbal adverse events from spontaneous reporting systems using taxonomic name resolution approach. Bioinform. Biol. Insights.

[9] Khaleel MA, Khan AH, Ghadzi SMS, Adnan AS, Abdallah QM (2022). A standardized dataset of a spontaneous adverse event reporting system. Healthcare (Basel).

[10] Sharma V, Restrepo MI, Sarkar IN (2019). Solr-Plant: efficient extraction of plant names from text. BMC Bioinformatics.

[11] difflib — Helpers for computing deltas. *Python documentation* https://docs.python.org/3/library/difflib.html.

[12] Chicco D, Cartwright H (2021). Siamese Neural Networks: An Overview. Artificial Neural Networks.

[13] Koch, G., Zemel, R. & Salakhutdinov, R. Siamese Neural Networks for One-shot Image Recognition. 8.

[14] Birer-Williams C (2020). A new data repository for pharmacokinetic natural product-drug interactions: From chemical characterization to clinical studies. Drug Metab. Dispos.

[15] Team, K. Keras documentation: Embedding layer. https://keras.io/api/layers/core_layers/embedding/.

[16] Hochreiter S, Schmidhuber J (1997). Long short-term memory. Neural Comput..

[17] tf.edit_distance | TensorFlow v2.11.0. https://www.tensorflow.org/api_docs/python/tf/edit_distance.

[18] Craswell N, Liu L, Özsu MT (2009). Mean Reciprocal Rank. Encyclopedia of Database Systems.

[19] Smith, T., Majid, F., Eckl, V. & Reynolds, C. M. Herbal Supplement Sales in US Increase by Record-Breaking 17.3% in 2020. 14.

[20] McHugh ML (2012). Interrater reliability: The kappa statistic. Biochem. Med. (Zagreb).

[21] Banda JM (2016). A curated and standardized adverse drug event resource to accelerate drug safety research. Sci. Data.

