# Supplementary material for: Broadening the capture of natural products mentioned in FAERS using fuzzy string-matching and a Siamese neural network
Source: Sci Rep. 2024 Jan 13;14:1272. doi: 10.1038/s41598-023-51004-4 (PMC10787736; doi:10.1038/s41598-023-51004-4)
Supplement: Supplementary file 1 — Supplementary Information. [file 41598_2023_51004_MOESM1_ESM.docx]

Appendix:

## Natural Product Name Variations Identified in FAERS Reports by Siamese Model:

| **Query NP name** | **Identified drug string** |
| --- | --- |
| ACAI Synonyms: ['EUTERPE OLERACEA', 'ACAI'] | ACIA |
| AESCULUS HIPPOCASTANUM Synonyms: ['AESCULUS HIPPOCASTANUM', 'HORSECHESTNUT'] | HORSECHESTNUT EXTRACT |
| AESCULUS HIPPOCASTANUM Synonyms: ['AESCULUS HIPPOCASTANUM', 'HORSECHESTNUT'] | AESCINTEVA ESCINUM |
| ALOE VERA Synonyms: ['ALOE VERA'] | ALOE VERA ALOE VERA |
| ALOE VERA Synonyms: ['ALOE VERA'] | ALOE VERA GEL ALOE VERA |
| ALOE VERA Synonyms: ['ALOE VERA'] | ALOEVERA EXTRA |
| ASHWAGANDA Synonyms: ['ASHWAGANDA', 'WITHANIA SOMNIFERA'] | ASHWAGHANDA AND MAGNESIUM |
| ASHWAGANDA Synonyms: ['ASHWAGANDA', 'WITHANIA SOMNIFERA'] | ASHWAGANDAFOR ADRENAL SUPPORT |
| ASHWAGANDA Synonyms: ['ASHWAGANDA', 'WITHANIA SOMNIFERA'] | ASHWAGANDHA MAGNESIUM |
| BEET ROOT Synonyms: ['BEET ROOT', 'BETA VULGARIS'] | BEETROOT EXTRACT |
| BETA VULGARIS Synonyms: ['BEET ROOT', 'BETA VULGARIS'] | B BEET JUICE |
| BETA VULGARIS Synonyms: ['BEET ROOT', 'BETA VULGARIS'] | BEET |
| BETA VULGARIS Synonyms: ['BEET ROOT', 'BETA VULGARIS'] | BEET LEAF |
| BETA VULGARIS Synonyms: ['BEET ROOT', 'BETA VULGARIS'] | BEET PILLS |
| BLACK COHOSH Synonyms: ['ACTAEA RACEMOSA', 'BLACK COHOSH'] | BLACK COHOSH CIMICIFUGA RACEMOSA CIMICIFUGA RACEMOSA |
| BLACK COHOSH Synonyms: ['ACTAEA RACEMOSA', 'BLACK COHOSH'] | BLACK COHOSH CIMICIFUGA RACEMOSA |
| BOSWELLIA Synonyms: ['BOSWELFIA SERRATA', 'BOSWELLIA'] | BOSWELLIACURCUMIN |
| CANNABIS SATIVA Synonyms: ['HEMP EXTRACT', 'CANNABIS SATIVA'] | CANNABISCANNABIS CANNABIS SATIVA |
| CANNABIS SATIVA Synonyms: ['HEMP EXTRACT', 'CANNABIS SATIVA'] | CANNABISCANNABIS SATIVA L |
| CHAMOMILE Synonyms: ['CHAMOMILE', 'MATRICARIA CHAMOMILLA'] | CHAMOMILE TEA OCCASIONALLY |
| CHELIDONIUM MAJUS Synonyms: ['SWALLOWWORT', 'CHELIDONIUM MAJUS'] | CHELIDONIUM CURCUMA TARAXACUM STANNO CULTUM |
| CHLORELLA Synonyms: ['CHLORELLA VULGARIS', 'CHLORELLA'] | CHLORELLA CHLORELLA |
| CHLORELLA VULGARIS Synonyms: ['CHLORELLA VULGARIS', 'CHLORELLA'] | CHLORELLA GROWTH FACTOR |
| CHLORELLA VULGARIS Synonyms: ['CHLORELLA VULGARIS', 'CHLORELLA'] | CHLORELA |
| CINNAMON Synonyms: ['CINNAMOMUM VERUM', 'CINNAMOMUM CASSIA', 'CINNAMON'] | CINAMMON CAPSULE |
| CINNAMON Synonyms: ['CINNAMOMUM VERUM', 'CINNAMOMUM CASSIA', 'CINNAMON'] | CIMMINON |
| CINNAMON Synonyms: ['CINNAMOMUM VERUM', 'CINNAMOMUM CASSIA', 'CINNAMON'] | COQ WITH CINAMON |
| CINNAMON Synonyms: ['CINNAMOMUM VERUM', 'CINNAMOMUM CASSIA', 'CINNAMON'] | CINNIOM |
| CRANBERRY Synonyms: ['VACCINIUM MACROCARPON', 'CRANBERRY'] | VACCINIUM MACROCARPON VACCINIUM MACROCARPON |
| CRANBERRY Synonyms: ['VACCINIUM MACROCARPON', 'CRANBERRY'] | CRANBERY CAPSULE VACCINIUM MACROCARPON |
| CRANBERRY Synonyms: ['VACCINIUM MACROCARPON', 'CRANBERRY'] | CRANBEROLA CYSCONTROL |
| CRANBERRY Synonyms: ['VACCINIUM MACROCARPON', 'CRANBERRY'] | CRANBERRRY SUPPLEMENT |
| CRANBERRY Synonyms: ['VACCINIUM MACROCARPON', 'CRANBERRY'] | CRANBERRYASCORBIC ACID |
| CURCUMA JONGA Synonyms: ['TURMERIC', 'CURCUMA JONGA'] | CURCUMA LONGA CURCUMA LONGA |
| CURCUMA JONGA Synonyms: ['TURMERIC', 'CURCUMA JONGA'] | TUMERIC CURCUMA LONGA UNKNOWN |
| CURCUMA JONGA Synonyms: ['TURMERIC', 'CURCUMA JONGA'] | CURCUMIN TURMERIC |
| CURCUMA JONGA Synonyms: ['TURMERIC', 'CURCUMA JONGA'] | CURCUMIN WMERIVA |
| CURCUMA JONGA Synonyms: ['TURMERIC', 'CURCUMA JONGA'] | CURCUMINTURMERIC |
| CURCUMA JONGA Synonyms: ['TURMERIC', 'CURCUMA JONGA'] | CURCUMA TUMERIC |
| CURCUMA JONGA Synonyms: ['TURMERIC', 'CURCUMA JONGA'] | TURMERIC CURCUMIN CURCUMA LONGA |
| CURCUMA JONGA Synonyms: ['TURMERIC', 'CURCUMA JONGA'] | CURCUMIN ALPHA |
| CURCUMA JONGA Synonyms: ['TURMERIC', 'CURCUMA JONGA'] | CURCUMEVAIL |
| ECHINACEA PURPUREA Synonyms: ['ECHINACEA PURPUREA', 'ECHINACEAPURPLE CONEFLOWER'] | ZINCECHINACEA |
| ECHINACEA PURPUREA Synonyms: ['ECHINACEA PURPUREA', 'ECHINACEAPURPLE CONEFLOWER'] | ECHINACEA ECHINACHEA PURPUREA ECHINACHEA PURPUREA |
| ECHINACEA PURPUREA Synonyms: ['ECHINACEA PURPUREA', 'ECHINACEAPURPLE CONEFLOWER'] | ECHINACEA ECHINACEA PURPURA ECHINACEA PURPURA |
| ECHINACEA Synonyms: ['ECHINACEA', 'ECHINACEA ANGUSTIFOLIA'] | ECHINAGARD |
| ECHINACEA Synonyms: ['ECHINACEA', 'ECHINACEA ANGUSTIFOLIA'] | ECHINACEA ECHINACEA PURPURA ECHINACEA PURPURA |
| ECHINACEA Synonyms: ['ECHINACEA', 'ECHINACEA ANGUSTIFOLIA'] | ECHINACEA ECHINACEA SPP TA |
| ECHINACEA Synonyms: ['ECHINACEA', 'ECHINACEA ANGUSTIFOLIA'] | ECHINACEA ECHINACEA SPP |
| ECHINACEA Synonyms: ['ECHINACEA', 'ECHINACEA ANGUSTIFOLIA'] | ENCINACHIA AMERICA |
| ECHINACEA Synonyms: ['ECHINACEA', 'ECHINACEA ANGUSTIFOLIA'] | ZINCVITAMIN CENCHINACEA |
| ECHINACEA Synonyms: ['ECHINACEA', 'ECHINACEA ANGUSTIFOLIA'] | ECHINACEA ECHINACEA ANGUSTIFOLIA |
| ECHINACEA Synonyms: ['ECHINACEA', 'ECHINACEA ANGUSTIFOLIA'] | ECHINACEA ECHINACEA |
| ECHINACEA Synonyms: ['ECHINACEA', 'ECHINACEA ANGUSTIFOLIA'] | ECHILNACEA ECHINACEA ANGUSTIFOLIA |
| ECHINACEA Synonyms: ['ECHINACEA', 'ECHINACEA ANGUSTIFOLIA'] | ECHINACEA ECHINACEA PURPEA |
| ECHINACEAPURPLE CONEFLOWER Synonyms: ['ECHINACEA PURPUREA', 'ECHINACEAPURPLE CONEFLOWER'] | ECHINECEAECHINACEA COMPLEX |
| ELDERBERRY Synonyms: ['ELDERBERRY', 'SAMBUCUS NIGRA'] | ELDERBERRY SAMBUCUS NIGRA |
| ELDERBERRY Synonyms: ['ELDERBERRY', 'SAMBUCUS NIGRA'] | ELDERBERRY SUPPLEMENT |
| ELDERBERRY Synonyms: ['ELDERBERRY', 'SAMBUCUS NIGRA'] | ELDERBERRY CONCENTRATE |
| ELDERBERRY Synonyms: ['ELDERBERRY', 'SAMBUCUS NIGRA'] | ELDERBERRYSAMBUCUS NIGRA |
| ELDERBERRY Synonyms: ['ELDERBERRY', 'SAMBUCUS NIGRA'] | ELDERBERRY SYRUP BRAND HONEY GARDENS |
| ELDERBERRY Synonyms: ['ELDERBERRY', 'SAMBUCUS NIGRA'] | SAMBUCUS BLACK ELDERBERRY MG |
| FENNEL Synonyms: ['FOENICULUM VULGARE', 'FENNEL'] | FENNEL FOENICULUM VULGARE SEED |
| FENNEL Synonyms: ['FOENICULUM VULGARE', 'FENNEL'] | SENNAFENNEL |
| FENUGREEK Synonyms: ['TRIGONELFA FOENUM', 'FENUGREEK'] | FRNNEGRUEK |
| FLAX SEED Synonyms: ['LINUM USITATISSIMUM', 'FLAX SEED'] | FLAX SEED OIL PILL |
| FOENICULUM VULGARE Synonyms: ['FOENICULUM VULGARE', 'FENNEL'] | FENNEL |
| GARCINIA GUMMI Synonyms: ['GARCINIA GUMMI', 'GARCINIA'] | GARCINA CAMBOGA |
| GARCINIA GUMMI Synonyms: ['GARCINIA GUMMI', 'GARCINIA'] | GARCINIA CAMBOGIA COMP |
| GARCINIA GUMMI Synonyms: ['GARCINIA GUMMI', 'GARCINIA'] | GARCINIA CAMBOGIA GARCINIA GUMMIGUTTA |
| GARCINIA Synonyms: ['GARCINIA GUMMI', 'GARCINIA'] | GARCINIA CAMBOGIA COMP |
| GARCINIA Synonyms: ['GARCINIA GUMMI', 'GARCINIA'] | GARCINIA CAMBOGIA ORAL |
| GARCINIA Synonyms: ['GARCINIA GUMMI', 'GARCINIA'] | GARCINIA CAMBROGIA |
| GARCINIA Synonyms: ['GARCINIA GUMMI', 'GARCINIA'] | GARCINIA CAMBOGIA |
| GARCINIA Synonyms: ['GARCINIA GUMMI', 'GARCINIA'] | GARCINIA GAMBOGIA |
| GARCINIA Synonyms: ['GARCINIA GUMMI', 'GARCINIA'] | GARCINIA GUMMIGUTTA |
| GARCINIA Synonyms: ['GARCINIA GUMMI', 'GARCINIA'] | GARCINIA CAMBODIA |
| GARCINIA Synonyms: ['GARCINIA GUMMI', 'GARCINIA'] | GARCINIA EXTRACT |
| GINGER Synonyms: ['GINGER', 'ZINGIBER OFFICINALE'] | GINGER ZINGIBER OFFICINALE ROOT |
| GINKGO BILOBA Synonyms: ['GINKGO BILOBA', 'GINKGO'] | GINGKO LOBA GINKGO BILOBA |
| GINKGO BILOBA Synonyms: ['GINKGO BILOBA', 'GINKGO'] | GINKGO BILOBA GINGKO BILOBA |
| GINKGO BILOBA Synonyms: ['GINKGO BILOBA', 'GINKGO'] | GINKGO BILOBA GINKGO BILOBA |
| GINKGO BILOBA Synonyms: ['GINKGO BILOBA', 'GINKGO'] | GINKO BILOBA GINKGO BILOBA |
| GINKGO BILOBA Synonyms: ['GINKGO BILOBA', 'GINKGO'] | GINKOBILOBA GINKGO BILOBA |
| GINKGO BILOBA Synonyms: ['GINKGO BILOBA', 'GINKGO'] | GINKGO BILOBA GINKO BILOBA |
| GINKGO BILOBA Synonyms: ['GINKGO BILOBA', 'GINKGO'] | GINKGO BILOBA NGXGINKGO BILOBA |
| GINKGO BILOBA Synonyms: ['GINKGO BILOBA', 'GINKGO'] | GINKGO BILOBA KINKGO BILOBA |
| GINKGO BILOBA Synonyms: ['GINKGO BILOBA', 'GINKGO'] | GINKO BILOBAGINKGO BILOBA |
| GINKGO BILOBA Synonyms: ['GINKGO BILOBA', 'GINKGO'] | GINKGO BILOBAGINKGO BILOBA |
| GINKGO Synonyms: ['GINKGO BILOBA', 'GINKGO'] | GINKGO GINKGO BILBOA |
| GINKGO Synonyms: ['GINKGO BILOBA', 'GINKGO'] | GINKO GINKGO BILOBA |
| GINKGO Synonyms: ['GINKGO BILOBA', 'GINKGO'] | GINKGOGINKGO BILOBA |
| GINKGO Synonyms: ['GINKGO BILOBA', 'GINKGO'] | GINKGO GINKGO BILOBE |
| GINKGO Synonyms: ['GINKGO BILOBA', 'GINKGO'] | GINKGO GINKGO BILOBA |
| GINKGO Synonyms: ['GINKGO BILOBA', 'GINKGO'] | GINKO GINKO |
| GINKGO Synonyms: ['GINKGO BILOBA', 'GINKGO'] | GINKGO BILOBA FORMULATION UNKNOWN GINKGO BILOBA |
| GINKGO Synonyms: ['GINKGO BILOBA', 'GINKGO'] | GINGKOMIN GINKGO BILOBA |
| GINKGO Synonyms: ['GINKGO BILOBA', 'GINKGO'] | GINKGO BILOBA GINKO BILOBA |
| GINKGO Synonyms: ['GINKGO BILOBA', 'GINKGO'] | GINGKO GINKGO BILOBA |
| GINKGO Synonyms: ['GINKGO BILOBA', 'GINKGO'] | GINKGO BILOBA GINGKO BILOBA |
| GINKGO Synonyms: ['GINKGO BILOBA', 'GINKGO'] | GINKOBA GINKGO BILOBA |
| GOJI BERRY Synonyms: ['LYCIUM BARBARUM', 'GOJI BERRY'] | WOLFBERRIES |
| GRAPEFRUIT Synonyms: ['GRAPEFRUIT', 'CITRUS PARADISI'] | GRAPEFUIT SEED EXTRACT |
| GREEN TEA Synonyms: ['GREEN TEA', 'CAMELLIA SINENSIS'] | GREEN TREE EXTRACT |
| HEMP EXTRACT Synonyms: ['HEMP EXTRACT', 'CANNABIS SATIVA'] | MARIJUANA NOS CANNABIS SATIVA |
| HEMP EXTRACT Synonyms: ['HEMP EXTRACT', 'CANNABIS SATIVA'] | CANNABIS CANNIBIS SATIVA |
| HEMP EXTRACT Synonyms: ['HEMP EXTRACT', 'CANNABIS SATIVA'] | CANNABISCANNABIS CANNABIS SATIVA |
| HEMP EXTRACT Synonyms: ['HEMP EXTRACT', 'CANNABIS SATIVA'] | CANNABIS CANNABIS SATIVA |
| HEMP EXTRACT Synonyms: ['HEMP EXTRACT', 'CANNABIS SATIVA'] | CANNABISCANNABIS SATIVA |
| HYPERICUM PERFORATUM Synonyms: ['HYPERICUM PERFORATUM', 'ST JOHNSWORT'] | SAINT JOHNS WORT |
| HYPERICUM PERFORATUM Synonyms: ['HYPERICUM PERFORATUM', 'ST JOHNSWORT'] | ST JHONS WORT |
| HYPERICUM PERFORATUM Synonyms: ['HYPERICUM PERFORATUM', 'ST JOHNSWORT'] | ST JOHNS WORT |
| HYPERICUM PERFORATUM Synonyms: ['HYPERICUM PERFORATUM', 'ST JOHNSWORT'] | ST JOHN WORT |
| HYPERICUM PERFORATUM Synonyms: ['HYPERICUM PERFORATUM', 'ST JOHNSWORT'] | ST JOHNS WORT ST JOHNS WORT |
| HYPERICUM PERFORATUM Synonyms: ['HYPERICUM PERFORATUM', 'ST JOHNSWORT'] | ST JOHNS WORTH |
| HYPERICUM PERFORATUM Synonyms: ['HYPERICUM PERFORATUM', 'ST JOHNSWORT'] | ST JOHNSWORT |
| HYPERICUM PERFORATUM Synonyms: ['HYPERICUM PERFORATUM', 'ST JOHNSWORT'] | STJOHNS WORT |
| HYPERICUM PERFORATUM Synonyms: ['HYPERICUM PERFORATUM', 'ST JOHNSWORT'] | SHARON PREPARAT ST JOHNS WORT HYPERICUMPERFORATUM |
| KAVA Synonyms: ['PIPER METHYSTICUM', 'KAVA'] | KAVA KAVA |
| KAVA Synonyms: ['PIPER METHYSTICUM', 'KAVA'] | CAVA CAVA |
| KRATOM Synonyms: ['MITRAGYNA SPECIOSA', 'KRATOM'] | KATROM MITRAGYNA SPECIOSA |
| LINUM USITATISSIMUM Synonyms: ['LINUM USITATISSIMUM', 'FLAX SEED'] | FLAX SEED OIL PILL |
| LIONSTOOTH Synonyms: ['LIONSTOOTH', 'TARAXACUM OFFICINALE'] | TARAXACUM OFFICINALE TARAXACUM TARAXACUM OFFICINALE |
| MATRICARIA CHAMOMILLA Synonyms: ['CHAMOMILE', 'MATRICARIA CHAMOMILLA'] | CHAMOMILE |
| MILK THISTLE Synonyms: ['MILK THISTLE', 'SILYBUM MARIANUM'] | SILIBUM MARIANUM |
| MILK THISTLE Synonyms: ['MILK THISTLE', 'SILYBUM MARIANUM'] | MILK TRISTLE SILYBUM MARIANUM |
| MILK THISTLE Synonyms: ['MILK THISTLE', 'SILYBUM MARIANUM'] | MILKWEED THISTLE MILKWEED THISTLE MILKWEED THISTLE |
| MILK THISTLE Synonyms: ['MILK THISTLE', 'SILYBUM MARIANUM'] | MILK THRISTLE SILYBUM MARIANUM |
| MILK THISTLE Synonyms: ['MILK THISTLE', 'SILYBUM MARIANUM'] | MILK THISTLE CAPSULES |
| MILK THISTLE Synonyms: ['MILK THISTLE', 'SILYBUM MARIANUM'] | MILK THISTLE SILYBUM MARIANUM |
| MILK THISTLE Synonyms: ['MILK THISTLE', 'SILYBUM MARIANUM'] | MILK THISTLE MILE THISTLE |
| MILK THISTLE Synonyms: ['MILK THISTLE', 'SILYBUM MARIANUM'] | MILK THISTLE SEED EXTRACT SILYBUM MARIANUM |
| MILK THISTLE Synonyms: ['MILK THISTLE', 'SILYBUM MARIANUM'] | MILK THISTLE SEED SILYBUM MARIANUM |
| MILK THISTLE Synonyms: ['MILK THISTLE', 'SILYBUM MARIANUM'] | MILK THISTLE CAPSULE |
| MIRACLEFRUIT Synonyms: ['GYMNEMA SYLVESTRE', 'MIRACLEFRUIT'] | GYMMEMA SYLVESTRE LEAF ASTTRACT |
| MORINGA OLEIFERA Synonyms: ['MORINGA', 'MORINGA OLEIFERA'] | MORINGA |
| MORINGA Synonyms: ['MORINGA', 'MORINGA OLEIFERA'] | MORINGA MORINGA OLEIFERA |
| MORINGA Synonyms: ['MORINGA', 'MORINGA OLEIFERA'] | MORINGA PREMIUM |
| OLEA EUROPAEA Synonyms: ['OLIVE LEAF', 'OLEA EUROPAEA'] | OLIVE LEAF |
| OLEA EUROPAEA Synonyms: ['OLIVE LEAF', 'OLEA EUROPAEA'] | OLIVE LEAF COMPLEX |
| OLIVE LEAF Synonyms: ['OLIVE LEAF', 'OLEA EUROPAEA'] | OLEA EUROPAE |
| OLIVE LEAF Synonyms: ['OLIVE LEAF', 'OLEA EUROPAEA'] | OLEA EUROPAEA LEAF |
| OLIVE LEAF Synonyms: ['OLIVE LEAF', 'OLEA EUROPAEA'] | OLIVE LEAF OLEA EUROPAEA LEAF |
| OLIVE LEAF Synonyms: ['OLIVE LEAF', 'OLEA EUROPAEA'] | OLIVE LEAVES EXTRACT |
| OLIVE LEAF Synonyms: ['OLIVE LEAF', 'OLEA EUROPAEA'] | OLIVE LEAVES EXTRACT OLIVE LEAVES EXTRACT |
| OLIVE LEAF Synonyms: ['OLIVE LEAF', 'OLEA EUROPAEA'] | OLIVE OIL OLEA EUROPAEA OIL |
| OLIVE LEAF Synonyms: ['OLIVE LEAF', 'OLEA EUROPAEA'] | OLIVE OIL OLEA EUROPEA |
| OLIVE LEAF Synonyms: ['OLIVE LEAF', 'OLEA EUROPAEA'] | OLIVE OIL OLEA EUROPEA OIL |
| OREGANO Synonyms: ['ORIGANUM VULGARE', 'OREGANO'] | ORIGAN ESSENTIAL OIL BY MOUTH DILUATE |
| OREGANO Synonyms: ['ORIGANUM VULGARE', 'OREGANO'] | OREGNO OIL |
| OREGANO Synonyms: ['ORIGANUM VULGARE', 'OREGANO'] | ORIGANUM SPP ESSENTIAL OIL |
| ORYZA SATIVA Synonyms: ['ORYZA SATIVA', 'RED YEAST RICE'] | RED YEAST CAB |
| ORYZA SATIVA Synonyms: ['ORYZA SATIVA', 'RED YEAST RICE'] | RED YEAST CAP |
| ORYZA SATIVA Synonyms: ['ORYZA SATIVA', 'RED YEAST RICE'] | RED YEAST EXTRACT |
| ORYZA SATIVA Synonyms: ['ORYZA SATIVA', 'RED YEAST RICE'] | RED YEAST RIC |
| ORYZA SATIVA Synonyms: ['ORYZA SATIVA', 'RED YEAST RICE'] | RED YEAST RISE |
| PIPER METHYSTICUM Synonyms: ['PIPER METHYSTICUM', 'KAVA'] | CAVA CAVA |
| PIPER METHYSTICUM Synonyms: ['PIPER METHYSTICUM', 'KAVA'] | KAVA |
| PIPER METHYSTICUM Synonyms: ['PIPER METHYSTICUM', 'KAVA'] | KAVA KAVA |
| RED YEAST RICE Synonyms: ['ORYZA SATIVA', 'RED YEAST RICE'] | RED YEAST NOS RED YEAST NOS |
| REISHI Synonyms: ['REISHI', 'GANODERMA LUCIDUM'] | REISHI SHIITAKE |
| REISHI Synonyms: ['REISHI', 'GANODERMA LUCIDUM'] | RED REISHI MUSHROOM |
| RHODIOLA ROSEA Synonyms: ['RHODIOLA ROSEA', 'RHODIOLA'] | RHODIOLA ROSEA MAGNESIUMRHODIOLA ROSEA ROOTTHIAMINE HYDROCHLORIDE |
| SAMBUCUS NIGRA Synonyms: ['ELDERBERRY', 'SAMBUCUS NIGRA'] | SAMBUCUS BLACK ELDERBERRY MG |
| SAMBUCUS NIGRA Synonyms: ['ELDERBERRY', 'SAMBUCUS NIGRA'] | SAMBUCOLELDERBERRY |
| SAMBUCUS NIGRA Synonyms: ['ELDERBERRY', 'SAMBUCUS NIGRA'] | SAMBUCOL BLACK ELDERBERRY |
| SAMBUCUS NIGRA Synonyms: ['ELDERBERRY', 'SAMBUCUS NIGRA'] | EDELBERRY ELDERBERRY |
| SAMBUCUS NIGRA Synonyms: ['ELDERBERRY', 'SAMBUCUS NIGRA'] | ELDERBERRY |
| SAMBUCUS NIGRA Synonyms: ['ELDERBERRY', 'SAMBUCUS NIGRA'] | ELDERBERRY CONCENTRATE |
| SAMBUCUS NIGRA Synonyms: ['ELDERBERRY', 'SAMBUCUS NIGRA'] | SAMBUCAI ELDERBERRY |
| SCRUBPALMETTO Synonyms: ['SCRUBPALMETTO', 'SERENOA REPENS'] | SARCOSAN BENZYL BENZOATESERENOA REPENS |
| SCRUBPALMETTO Synonyms: ['SCRUBPALMETTO', 'SERENOA REPENS'] | SAW PALMETO SAW PALMETO |
| SCRUBPALMETTO Synonyms: ['SCRUBPALMETTO', 'SERENOA REPENS'] | HERBAL SUPPLEMENT PALMETTO |
| SCRUBPALMETTO Synonyms: ['SCRUBPALMETTO', 'SERENOA REPENS'] | SAW PALMENTTO SAW PALMETT |
| SENNA ALEXANDRINA Synonyms: ['SENNA ALEXANDRINA', 'SENNA'] | SENNAS DOCUSATE SODIUMSENNA ALEXANDRINA |
| SENNA ALEXANDRINA Synonyms: ['SENNA ALEXANDRINA', 'SENNA'] | SENNAS DOCUSATE SODIUM SENNA ALEXANDRINA |
| SENNA ALEXANDRINA Synonyms: ['SENNA ALEXANDRINA', 'SENNA'] | SENNALAXS |
| SENNA ALEXANDRINA Synonyms: ['SENNA ALEXANDRINA', 'SENNA'] | SENNA TABLETS SENNA ALEXANDRINA TA |
| SENNA ALEXANDRINA Synonyms: ['SENNA ALEXANDRINA', 'SENNA'] | SENNA AND LAXATIVE SENNA ALEXANDRINA |
| SENNA ALEXANDRINA Synonyms: ['SENNA ALEXANDRINA', 'SENNA'] | SENNA VITAMIN D |
| SENNA ALEXANDRINA Synonyms: ['SENNA ALEXANDRINA', 'SENNA'] | SENOKOTSENNA ALSEXANDRINA SENNA ALSEXANDRINA |
| SENNA ALEXANDRINA Synonyms: ['SENNA ALEXANDRINA', 'SENNA'] | SENOKOTUSASENNA SENNA ALEXANDRINA |
| SENNA ALEXANDRINA Synonyms: ['SENNA ALEXANDRINA', 'SENNA'] | SENOKOT SENNA ALEXANDRINA SENNA ALEXANDRINA |
| SENNA ALEXANDRINA Synonyms: ['SENNA ALEXANDRINA', 'SENNA'] | SENNA SENNA ALEXANDRINA UNKNOWN |
| SENNA ALEXANDRINA Synonyms: ['SENNA ALEXANDRINA', 'SENNA'] | SENNA LEAF AND SENNA POD |
| SENNA Synonyms: ['SENNA ALEXANDRINA', 'SENNA'] | SENN |
| SENNA Synonyms: ['SENNA ALEXANDRINA', 'SENNA'] | SEENAS |
| SENNA Synonyms: ['SENNA ALEXANDRINA', 'SENNA'] | XENNA |
| SENNA Synonyms: ['SENNA ALEXANDRINA', 'SENNA'] | SEENA |
| SERENOA REPENS Synonyms: ['SCRUBPALMETTO', 'SERENOA REPENS'] | SAW PALMENTTO SAW PALMETT |
| SERENOA REPENS Synonyms: ['SCRUBPALMETTO', 'SERENOA REPENS'] | SAW PALMETO SAW PALMETO |
| SILYBUM MARIANUM Synonyms: ['MILK THISTLE', 'SILYBUM MARIANUM'] | MILK THISTLE SELYBUM MARIANUM |
| SILYBUM MARIANUM Synonyms: ['MILK THISTLE', 'SILYBUM MARIANUM'] | MILK THISTLE SEED SILYBUM MARIANUM |
| SILYBUM MARIANUM Synonyms: ['MILK THISTLE', 'SILYBUM MARIANUM'] | MILK THISTLE MILE THISTLE |
| SILYBUM MARIANUM Synonyms: ['MILK THISTLE', 'SILYBUM MARIANUM'] | MILK THISTLE SILYBUM MARIANUM SILYBUM MARIANUM |
| SILYBUM MARIANUM Synonyms: ['MILK THISTLE', 'SILYBUM MARIANUM'] | SILYBUM MARIANUM SILYBUM MARIANUM |
| SILYBUM MARIANUM Synonyms: ['MILK THISTLE', 'SILYBUM MARIANUM'] | MILKWEED THISTLE MILKWEED THISTLE MILKWEED THISTLE |
| ST JOHNSWORT Synonyms: ['HYPERICUM PERFORATUM', 'ST JOHNSWORT'] | ST JOHNS WORT ST JOHNS WORT |
| ST JOHNSWORT Synonyms: ['HYPERICUM PERFORATUM', 'ST JOHNSWORT'] | ST JOHNS WORT EXTRACT |
| ST JOHNSWORT Synonyms: ['HYPERICUM PERFORATUM', 'ST JOHNSWORT'] | ST JOHNS WORT HYPERICUM PERFORMATUM |
| STEVIA REBAUDIANA Synonyms: ['STEVIA REBAUDIANA', 'STEVIA'] | STEVA |
| STEVIA Synonyms: ['STEVIA REBAUDIANA', 'STEVIA'] | STEVIA STEVIA REBAUDIANA |
| TANACETUM PARTHENIUM Synonyms: ['TANACETUM PARTHENIUM', 'FEVERFEW'] | FEVERFEW |
| TARAXACUM OFFICINALE Synonyms: ['LIONSTOOTH', 'TARAXACUM OFFICINALE'] | TARAXACUM OFFICINALE TARAXACUM TARAXACUM OFFICINALE |
| TRITICUM AESTIVUM Synonyms: ['WHEAT GRASS', 'TRITICUM AESTIVUM'] | WHEAT |
| TURMERIC Synonyms: ['TURMERIC', 'CURCUMA JONGA'] | TURMERICCURCUMIN |
| TURMERIC Synonyms: ['TURMERIC', 'CURCUMA JONGA'] | TURMERIC CURCUMA LONGA |
| TURMERIC Synonyms: ['TURMERIC', 'CURCUMA JONGA'] | TURMERIC CURCUMA |
| TURMERIC Synonyms: ['TURMERIC', 'CURCUMA JONGA'] | TURMERIC TUMERIC |
| TURMERIC Synonyms: ['TURMERIC', 'CURCUMA JONGA'] | TUMERICBROMELAIN |
| TURMERIC Synonyms: ['TURMERIC', 'CURCUMA JONGA'] | CURCUMINTURMERIC |
| TURMERIC Synonyms: ['TURMERIC', 'CURCUMA JONGA'] | TUMERICCURCUMIN |
| TURMERIC Synonyms: ['TURMERIC', 'CURCUMA JONGA'] | KURKUMA CURCUMA LONGA |
| VACCINIUM MACROCARPON Synonyms: ['VACCINIUM MACROCARPON', 'CRANBERRY'] | CRAN BERRY |
| VALERIAN Synonyms: ['VALERIAN', 'VALERIANA OFFICINALIS'] | VALERIAN VALERIAN |
| VALERIAN Synonyms: ['VALERIAN', 'VALERIANA OFFICINALIS'] | VALERIAN ROOT |
| VALERIAN Synonyms: ['VALERIAN', 'VALERIANA OFFICINALIS'] | VALERIAN VALERIANA OFFICINALIS |
| VALERIAN Synonyms: ['VALERIAN', 'VALERIANA OFFICINALIS'] | VALERIAAN VALERIAAN |
| VALERIAN Synonyms: ['VALERIAN', 'VALERIANA OFFICINALIS'] | VALERIANA OFFICIALS VALERIANA OFFICINALIS |
| VALERIAN Synonyms: ['VALERIAN', 'VALERIANA OFFICINALIS'] | VALERIANA OFFICINALIS VALERIANA OFFICINALIS |
| VALERIAN Synonyms: ['VALERIAN', 'VALERIANA OFFICINALIS'] | VALERIANA OFFICINALISVALERIANA OFFICINALIS |
| VALERIAN Synonyms: ['VALERIAN', 'VALERIANA OFFICINALIS'] | VALERIAN HERBAL CAPSULES |
| VALERIAN Synonyms: ['VALERIAN', 'VALERIANA OFFICINALIS'] | VALERIANE RADIX |
| VALERIAN Synonyms: ['VALERIAN', 'VALERIANA OFFICINALIS'] | VALERIANVALERIANA OFFICINALIS |
| VALERIAN Synonyms: ['VALERIAN', 'VALERIANA OFFICINALIS'] | VALERIN JAPAN |
| VALERIAN Synonyms: ['VALERIAN', 'VALERIANA OFFICINALIS'] | VALERIN MAX |
| VALERIAN Synonyms: ['VALERIAN', 'VALERIANA OFFICINALIS'] | VALERIN VALERIANA OFFICINALIS |
| VALERIAN Synonyms: ['VALERIAN', 'VALERIANA OFFICINALIS'] | VALVERDE BALDRIAN |
| VALERIAN Synonyms: ['VALERIAN', 'VALERIANA OFFICINALIS'] | VALERIANA OFFICINALIS VALRIANA OFFICINALIS |
| VALERIAN Synonyms: ['VALERIAN', 'VALERIANA OFFICINALIS'] | VALERIANAE COMP |
| VALERIANA OFFICINALIS Synonyms: ['VALERIAN', 'VALERIANA OFFICINALIS'] | VALERIANA OFFICIALS VALERIANA OFFICINALIS |
| VALERIANA OFFICINALIS Synonyms: ['VALERIAN', 'VALERIANA OFFICINALIS'] | VALERIANA EXTRACT |
| VALERIANA OFFICINALIS Synonyms: ['VALERIAN', 'VALERIANA OFFICINALIS'] | VALERIANA OFFICINALIS VALRIANA OFFICINALIS |
| VALERIANA OFFICINALIS Synonyms: ['VALERIAN', 'VALERIANA OFFICINALIS'] | VALERIANA ALFA |
| VALERIANA OFFICINALIS Synonyms: ['VALERIAN', 'VALERIANA OFFICINALIS'] | VALERIANA OFFICINALISVALERIANA OFFICINALIS |
| VALERIANA OFFICINALIS Synonyms: ['VALERIAN', 'VALERIANA OFFICINALIS'] | VALERIANA |
| VALERIANA OFFICINALIS Synonyms: ['VALERIAN', 'VALERIANA OFFICINALIS'] | VALVERDE BALDRIAN HOPFEN VALERIANA OFFICINALIS |
| VALERIANA OFFICINALIS Synonyms: ['VALERIAN', 'VALERIANA OFFICINALIS'] | VALERIAN TINCTURE VALERIANA OFFICINALIS TINCTURE |
| VALERIANA OFFICINALIS Synonyms: ['VALERIAN', 'VALERIANA OFFICINALIS'] | VALERIAN HERBAL CAPSULES |
| VALERIANA OFFICINALIS Synonyms: ['VALERIAN', 'VALERIANA OFFICINALIS'] | VALERIAAN VALERIAAN |
| VALERIANA OFFICINALIS Synonyms: ['VALERIAN', 'VALERIANA OFFICINALIS'] | VALERIANA OFFICINALIS VALERIANA OFFICINALIS |
| VALERIANA OFFICINALIS Synonyms: ['VALERIAN', 'VALERIANA OFFICINALIS'] | VALERIAAN |
| VALERIANA OFFICINALIS Synonyms: ['VALERIAN', 'VALERIANA OFFICINALIS'] | VALERIAN VALERIAN |
| WHEAT GRASS Synonyms: ['WHEAT GRASS', 'TRITICUM AESTIVUM'] | WHEAT |
| ACAI Synonyms: ['EUTERPE OLERACEA', 'ACAI'] | ACIA |
|  |  |

## Natural Product Name Variations Identified in FAERS Reports by Gestalt Pattern Matching:

| **Query NP name** | **Identified drug string** |
| --- | --- |
| AESCULUS HIPPOCASTANUM Synonyms: ['AESCULUS HIPPOCASTANUM', 'HORSECHESTNUT'] | RUTOSIDEAESCULUS HIPPOCASTANUM SEED |
| AESCULUS HIPPOCASTANUM Synonyms: ['AESCULUS HIPPOCASTANUM', 'HORSECHESTNUT'] | AESCULUS HIPPOCASTANUM EXTRACTTHIAMINE HYDROCHLORIDE |
| AESCULUS HIPPOCASTANUM Synonyms: ['AESCULUS HIPPOCASTANUM', 'HORSECHESTNUT'] | AESCULUS HIPPOCASTANUM SEED WMIROTONRUTOSID |
| AESCULUS HIPPOCASTANUM Synonyms: ['AESCULUS HIPPOCASTANUM', 'HORSECHESTNUT'] | VENCOUR TRIPLEX RUTOSIDEAESCULUSHIPPOCASTANUM L MIROT |
| AESCULUS HIPPOCASTANUM Synonyms: ['AESCULUS HIPPOCASTANUM', 'HORSECHESTNUT'] | VENOSIN AESCULUS HIPPOCASTANUM EXTRACT |
| ALOE VERA Synonyms: ['ALOE VERA'] | NACLALOE VERA |
| ALOE VERA Synonyms: ['ALOE VERA'] | ALOE VERA GEL |
| ALOE VERA Synonyms: ['ALOE VERA'] | ALOE VERA LOTION |
| ALOE VERA Synonyms: ['ALOE VERA'] | ALOE VERAMENTHOL |
| ALOE VERA Synonyms: ['ALOE VERA'] | ALOES EXTRACT |
| ALOE VERA Synonyms: ['ALOE VERA'] | ELOVERA |
| ALOE VERA Synonyms: ['ALOE VERA'] | ALOEVESTA |
| ALOE VERA Synonyms: ['ALOE VERA'] | COMFREYALOE VERA |
| ALOE VERA Synonyms: ['ALOE VERA'] | DRINK ALOE VERA |
| APPLE CIDER VINEGAR Synonyms: ['APPLE CIDER VINEGAR', 'MALUS DOMESTICA', 'MALUS PUMILA'] | APPLE ACID |
| APPLE CIDER VINEGAR Synonyms: ['APPLE CIDER VINEGAR', 'MALUS DOMESTICA', 'MALUS PUMILA'] | CIGAR VINEGAR |
| ASHWAGANDA Synonyms: ['ASHWAGANDA', 'WITHANIA SOMNIFERA'] | ASHWAGANDA SUPPLEMENT |
| ASHWAGANDA Synonyms: ['ASHWAGANDA', 'WITHANIA SOMNIFERA'] | ASHWAGANDHA MAGNESIUM |
| BARLEY GRASS Synonyms: ['HORDEUM VULGARE', 'BARLEY GRASS'] | NATURES WAY BARLEY GRASS |
| BEET ROOT Synonyms: ['BEET ROOT', 'BETA VULGARIS'] | BEETROOT JUICE |
| BEET ROOT Synonyms: ['BEET ROOT', 'BETA VULGARIS'] | BEET ROOT POWDER |
| BEET ROOT Synonyms: ['BEET ROOT', 'BETA VULGARIS'] | BEET ROOT COMPLEX |
| BLACK CHERRY Synonyms: ['PRUNUS SEROTINA', 'BLACK CHERRY'] | BLACK CHERRY CAPSULES |
| BLACK CHERRY Synonyms: ['PRUNUS SEROTINA', 'BLACK CHERRY'] | BLACK CHERRY CASPULE |
| BLACK CHERRY Synonyms: ['PRUNUS SEROTINA', 'BLACK CHERRY'] | VITAMIN BLACK CHERRY |
| BLACK COHOSH Synonyms: ['ACTAEA RACEMOSA', 'BLACK COHOSH'] | BLACK COHASH EXTRACT |
| BLACK COHOSH Synonyms: ['ACTAEA RACEMOSA', 'BLACK COHOSH'] | BLACK COHASH ROOT |
| BLACK COHOSH Synonyms: ['ACTAEA RACEMOSA', 'BLACK COHOSH'] | BLACK COHASH AND HERBS |
| BLACK COHOSH Synonyms: ['ACTAEA RACEMOSA', 'BLACK COHOSH'] | BLACK COHOSH SUPPLEMENT |
| BLACK COHOSH Synonyms: ['ACTAEA RACEMOSA', 'BLACK COHOSH'] | BLACK COHOSH CIMICIFUGA |
| BLACK COHOSH Synonyms: ['ACTAEA RACEMOSA', 'BLACK COHOSH'] | BLACK COHOSH EVENING |
| BLACK COHOSH Synonyms: ['ACTAEA RACEMOSA', 'BLACK COHOSH'] | BLACK OCHOSH CIMICIFUGA |
| BLACK COHOSH Synonyms: ['ACTAEA RACEMOSA', 'BLACK COHOSH'] | BLACK COHOSH TOPICAL |
| BLACK COHOSH Synonyms: ['ACTAEA RACEMOSA', 'BLACK COHOSH'] | BLACK COHOSH UNSPECIFIED |
| BLACK COHOSH Synonyms: ['ACTAEA RACEMOSA', 'BLACK COHOSH'] | COHOSH |
| BLACK COHOSH Synonyms: ['ACTAEA RACEMOSA', 'BLACK COHOSH'] | OTCHERBAL BLACK COHOSH |
| BLACK CUMIN Synonyms: ['BLACK CUMIN', 'NIGELLA SATIVA'] | BLACK SEED CUMIN OIL |
| BOSWELFIA SERRATA Synonyms: ['BOSWELFIA SERRATA', 'BOSWELLIA'] | NSI BOSWELLIA EXTRACT |
| BOSWELFIA SERRATA Synonyms: ['BOSWELFIA SERRATA', 'BOSWELLIA'] | GLUCOSAMINEBOSWELLIASERR |
| BOSWELFIA SERRATA Synonyms: ['BOSWELFIA SERRATA', 'BOSWELLIA'] | BOSWILLIA CURAMIN |
| BOSWELFIA SERRATA Synonyms: ['BOSWELFIA SERRATA', 'BOSWELLIA'] | BOSWELLIA TART CHERRY |
| BOSWELFIA SERRATA Synonyms: ['BOSWELFIA SERRATA', 'BOSWELLIA'] | BOSWELLIA COMPLEX |
| BOSWELFIA SERRATA Synonyms: ['BOSWELFIA SERRATA', 'BOSWELLIA'] | BOSWELLIA ACID |
| BOSWELFIA SERRATA Synonyms: ['BOSWELFIA SERRATA', 'BOSWELLIA'] | BOSWELLA CURAUM |
| BOSWELFIA SERRATA Synonyms: ['BOSWELFIA SERRATA', 'BOSWELLIA'] | BOSWELLIA SACRA NO PREF NAME |
| BOSWELLIA Synonyms: ['BOSWELFIA SERRATA', 'BOSWELLIA'] | TUMERICBOSWELLIA |
| BOSWELLIA Synonyms: ['BOSWELFIA SERRATA', 'BOSWELLIA'] | HERBAL BOSWELLIA |
| BOSWELLIA Synonyms: ['BOSWELFIA SERRATA', 'BOSWELLIA'] | BOSWALLA |
| CANNABIS SATIVA Synonyms: ['HEMP EXTRACT', 'CANNABIS SATIVA'] | CANNIBIS SATIVA TINCTURE |
| CANNABIS SATIVA Synonyms: ['HEMP EXTRACT', 'CANNABIS SATIVA'] | CANNABISCANNABIS SATIVA L |
| CANNABIS SATIVA Synonyms: ['HEMP EXTRACT', 'CANNABIS SATIVA'] | BLACK MAMBA CANNABIS SATIVA |
| CATSCLAW Synonyms: ['UNCARIA TOMENTOSA', 'CATSCLAW'] | CATS CALW BARK |
| CHAMOMILE Synonyms: ['CHAMOMILE', 'MATRICARIA CHAMOMILLA'] | CHAMOMILE TEA |
| CHAMOMILE Synonyms: ['CHAMOMILE', 'MATRICARIA CHAMOMILLA'] | VALERIANCHAMOMILE |
| CHAMOMILE Synonyms: ['CHAMOMILE', 'MATRICARIA CHAMOMILLA'] | MELATONINCHAMOMILE |
| CHLORELLA Synonyms: ['CHLORELLA VULGARIS', 'CHLORELLA'] | KING CHLORELLA |
| CHLORELLA Synonyms: ['CHLORELLA VULGARIS', 'CHLORELLA'] | CHLORETTA |
| CHLORELLA Synonyms: ['CHLORELLA VULGARIS', 'CHLORELLA'] | SUN CHLORELLA |
| CHLORELLA Synonyms: ['CHLORELLA VULGARIS', 'CHLORELLA'] | SUNCHLORELLA |
| CHLORELLA Synonyms: ['CHLORELLA VULGARIS', 'CHLORELLA'] | VIT C CHLORELLA |
| CHLORELLA Synonyms: ['CHLORELLA VULGARIS', 'CHLORELLA'] | SAN CHLORELLA |
| CHLORELLA VULGARIS Synonyms: ['CHLORELLA VULGARIS', 'CHLORELLA'] | CHLORELLA CHLORELLA SPP |
| CHLORELLA VULGARIS Synonyms: ['CHLORELLA VULGARIS', 'CHLORELLA'] | HLORELLA SEED |
| CHLORELLA VULGARIS Synonyms: ['CHLORELLA VULGARIS', 'CHLORELLA'] | CHORELLASPIRULINA |
| CHLORELLA VULGARIS Synonyms: ['CHLORELLA VULGARIS', 'CHLORELLA'] | CHLONELLA |
| CHLORELLA VULGARIS Synonyms: ['CHLORELLA VULGARIS', 'CHLORELLA'] | CHORELLA |
| CHLORELLA VULGARIS Synonyms: ['CHLORELLA VULGARIS', 'CHLORELLA'] | COQCHLORELLA |
| CHLORELLA VULGARIS Synonyms: ['CHLORELLA VULGARIS', 'CHLORELLA'] | SUNCHLORELLA |
| CINNAMOMUM CASSIA Synonyms: ['CINNAMOMUM VERUM', 'CINNAMOMUM CASSIA', 'CINNAMON'] | CINAMON CAPSULES |
| CINNAMOMUM VERUM Synonyms: ['CINNAMOMUM VERUM', 'CINNAMOMUM CASSIA', 'CINNAMON'] | CINNOMON BARK |
| CINNAMOMUM VERUM Synonyms: ['CINNAMOMUM VERUM', 'CINNAMOMUM CASSIA', 'CINNAMON'] | CINNAMONCHROMIUM SUPPLEMENT |
| CORDYCEPS Synonyms: ['CORDYCEPS', 'OPHIOCORDYCEPS SINENSIS'] | ORGANIC CORDYCEPS |
| CORDYCEPS Synonyms: ['CORDYCEPS', 'OPHIOCORDYCEPS SINENSIS'] | CORDYMAX CS |
| CORDYCEPS Synonyms: ['CORDYCEPS', 'OPHIOCORDYCEPS SINENSIS'] | CORDYCEPS MUSHROOM |
| CORDYCEPS Synonyms: ['CORDYCEPS', 'OPHIOCORDYCEPS SINENSIS'] | CORDYCEPS POWDER |
| CORDYCEPS Synonyms: ['CORDYCEPS', 'OPHIOCORDYCEPS SINENSIS'] | CORDYCEPS ACTIV |
| CRANBERRY Synonyms: ['VACCINIUM MACROCARPON', 'CRANBERRY'] | CRABERRY ESTERC |
| CRANBERRY Synonyms: ['VACCINIUM MACROCARPON', 'CRANBERRY'] | CRANBARRIER |
| CRANBERRY Synonyms: ['VACCINIUM MACROCARPON', 'CRANBERRY'] | CRANBRERRY EXTRACT |
| CRANBERRY Synonyms: ['VACCINIUM MACROCARPON', 'CRANBERRY'] | CARANEBERRY CAPS |
| CRANBERRY Synonyms: ['VACCINIUM MACROCARPON', 'CRANBERRY'] | GRANBERY CAP |
| CRANBERRY Synonyms: ['VACCINIUM MACROCARPON', 'CRANBERRY'] | CRANRX |
| CRANBERRY Synonyms: ['VACCINIUM MACROCARPON', 'CRANBERRY'] | CRANBERRRY EXTRACT |
| CRATAEGUS LAEVIGATA Synonyms: ['WOODLAND HAWTHORN', 'CRATAEGUS LAEVIGATA'] | HAWTHORN CRATEGUS LAEVIGATA CAPSULES |
| CURCUMA JONGA Synonyms: ['TURMERIC', 'CURCUMA JONGA'] | CURCUMIN |
| CURCUMA JONGA Synonyms: ['TURMERIC', 'CURCUMA JONGA'] | CUR CUMIN |
| CURCUMA JONGA Synonyms: ['TURMERIC', 'CURCUMA JONGA'] | CURCUMA DOMESTICA |
| CURCUMA JONGA Synonyms: ['TURMERIC', 'CURCUMA JONGA'] | CURCUMA LONGA RHIZOME |
| CURCUMA JONGA Synonyms: ['TURMERIC', 'CURCUMA JONGA'] | CURCUMA LONGA ROOT |
| CURCUMA JONGA Synonyms: ['TURMERIC', 'CURCUMA JONGA'] | CURCUMAX |
| CURCUMA JONGA Synonyms: ['TURMERIC', 'CURCUMA JONGA'] | CURCUMIA |
| CURCUMA JONGA Synonyms: ['TURMERIC', 'CURCUMA JONGA'] | CURCURMIN |
| CURCUMA JONGA Synonyms: ['TURMERIC', 'CURCUMA JONGA'] | CURCUMMIN |
| CURCUMA JONGA Synonyms: ['TURMERIC', 'CURCUMA JONGA'] | PROSTAMEN CURCUMA LONGA |
| ECHINACEA ANGUSTIFOLIA Synonyms: ['ECHINACEA', 'ECHINACEA ANGUSTIFOLIA'] | ECHINACEA AND GOLDEN SEAL |
| ECHINACEA ANGUSTIFOLIA Synonyms: ['ECHINACEA', 'ECHINACEA ANGUSTIFOLIA'] | ECHINACEAGOLDENSEAL |
| ECHINACEA ANGUSTIFOLIA Synonyms: ['ECHINACEA', 'ECHINACEA ANGUSTIFOLIA'] | ECHINACEA GOLDENSEAL G |
| ECHINACEA ANGUSTIFOLIA Synonyms: ['ECHINACEA', 'ECHINACEA ANGUSTIFOLIA'] | ECHINACEAPLUS |
| ECHINACEA ANGUSTIFOLIA Synonyms: ['ECHINACEA', 'ECHINACEA ANGUSTIFOLIA'] | ECHINACEA WGOLDENSEAL |
| ECHINACEA ANGUSTIFOLIA Synonyms: ['ECHINACEA', 'ECHINACEA ANGUSTIFOLIA'] | ECHINACEA AND GOLDENSEAL |
| ECHINACEA ANGUSTIFOLIA Synonyms: ['ECHINACEA', 'ECHINACEA ANGUSTIFOLIA'] | ECHINACEA GOLDEN SEAL |
| ECHINACEA ANGUSTIFOLIA Synonyms: ['ECHINACEA', 'ECHINACEA ANGUSTIFOLIA'] | ECHINACEA GOLDENSEAL |
| ECHINACEA ANGUSTIFOLIA Synonyms: ['ECHINACEA', 'ECHINACEA ANGUSTIFOLIA'] | ECHINACEAGOLDEN SEAL |
| ECHINACEA PURPUREA Synonyms: ['ECHINACEA PURPUREA', 'ECHINACEAPURPLE CONEFLOWER'] | ECHINACEA COMPOUND |
| ECHINACEA PURPUREA Synonyms: ['ECHINACEA PURPUREA', 'ECHINACEAPURPLE CONEFLOWER'] | ECHINACEA ECHINACEA PURPUREA ROOT |
| ECHINACEA PURPUREA Synonyms: ['ECHINACEA PURPUREA', 'ECHINACEAPURPLE CONEFLOWER'] | IMMUNEX ECHINACEA PURPUREA EXTRACT |
| ECHINACEA PURPUREA Synonyms: ['ECHINACEA PURPUREA', 'ECHINACEAPURPLE CONEFLOWER'] | ECHINACEA RATIOPHARM |
| ECHINACEA Synonyms: ['ECHINACEA', 'ECHINACEA ANGUSTIFOLIA'] | LIQUID ECHINACEA |
| ECHINACEA Synonyms: ['ECHINACEA', 'ECHINACEA ANGUSTIFOLIA'] | ECHINACEAPLUS |
| ECHINACEA Synonyms: ['ECHINACEA', 'ECHINACEA ANGUSTIFOLIA'] | ECHINACEA PLUS |
| ECHINACEA Synonyms: ['ECHINACEA', 'ECHINACEA ANGUSTIFOLIA'] | ECCHYNACEE |
| ECHINACEA Synonyms: ['ECHINACEA', 'ECHINACEA ANGUSTIFOLIA'] | HERBAL ECHINACEA |
| ECHINACEA Synonyms: ['ECHINACEA', 'ECHINACEA ANGUSTIFOLIA'] | AVOGEL ECHINACEA |
| ECHINACEAPURPLE CONEFLOWER Synonyms: ['ECHINACEA PURPUREA', 'ECHINACEAPURPLE CONEFLOWER'] | ECHINACEA COMPOUND |
| ECHINACEAPURPLE CONEFLOWER Synonyms: ['ECHINACEA PURPUREA', 'ECHINACEAPURPLE CONEFLOWER'] | ECHINACEA ECHINACEA COMPLEX |
| ECHINACEAPURPLE CONEFLOWER Synonyms: ['ECHINACEA PURPUREA', 'ECHINACEAPURPLE CONEFLOWER'] | ECHINACEA GOLDENSEAL |
| ELDERBERRY Synonyms: ['ELDERBERRY', 'SAMBUCUS NIGRA'] | ELDERBERRY SAMBUCUS |
| ELDERBERRY Synonyms: ['ELDERBERRY', 'SAMBUCUS NIGRA'] | SAMBUCCUS ELDERBERRY |
| ELDERBERRY Synonyms: ['ELDERBERRY', 'SAMBUCUS NIGRA'] | ZINC ELDERBERRY |
| ELDERBERRY Synonyms: ['ELDERBERRY', 'SAMBUCUS NIGRA'] | MULTI ELDERBERRY |
| ELDERBERRY Synonyms: ['ELDERBERRY', 'SAMBUCUS NIGRA'] | GAIA BLACK ELDERBERRY |
| ELDERBERRY Synonyms: ['ELDERBERRY', 'SAMBUCUS NIGRA'] | SAMBUCAI ELDERBERRY |
| ELDERBERRY Synonyms: ['ELDERBERRY', 'SAMBUCUS NIGRA'] | SAMBUCUS ELDERBERRY |
| ELDERBERRY Synonyms: ['ELDERBERRY', 'SAMBUCUS NIGRA'] | SAMBUCOLELDERBERRY |
| ELDERBERRY Synonyms: ['ELDERBERRY', 'SAMBUCUS NIGRA'] | OTC BLACK ELDERBERRY |
| EUTERPE OLERACEA Synonyms: ['EUTERPE OLERACEA', 'ACAI'] | ACAI BERRY EUTERPE OLERACEA FRUIT |
| EUTERPE OLERACEA Synonyms: ['EUTERPE OLERACEA', 'ACAI'] | ACAI EUTERPE OLERACEA FRUIT |
| EUTERPE OLERACEA Synonyms: ['EUTERPE OLERACEA', 'ACAI'] | EUTERPE OLERACEA FRUIT |
| EVENING PRIMROSE OIL Synonyms: ['EVENING PRIMROSE OIL', 'OENOTHERA BIENNIS'] | PRIMOSE OIL |
| EVENING PRIMROSE OIL Synonyms: ['EVENING PRIMROSE OIL', 'OENOTHERA BIENNIS'] | REGULAR PRIMROSE |
| EVENING PRIMROSE OIL Synonyms: ['EVENING PRIMROSE OIL', 'OENOTHERA BIENNIS'] | PRIMROST OIL |
| EVENING PRIMROSE OIL Synonyms: ['EVENING PRIMROSE OIL', 'OENOTHERA BIENNIS'] | EVEPRM OIL |
| EVENING PRIMROSE OIL Synonyms: ['EVENING PRIMROSE OIL', 'OENOTHERA BIENNIS'] | PRIMROSE SOFTGEL |
| EVENING PRIMROSE OIL Synonyms: ['EVENING PRIMROSE OIL', 'OENOTHERA BIENNIS'] | PRIMROSE HIP |
| EVENING PRIMROSE OIL Synonyms: ['EVENING PRIMROSE OIL', 'OENOTHERA BIENNIS'] | PRIMROSE FISH OIL |
| EVENING PRIMROSE OIL Synonyms: ['EVENING PRIMROSE OIL', 'OENOTHERA BIENNIS'] | EVEPREM OIL |
| EVENING PRIMROSE OIL Synonyms: ['EVENING PRIMROSE OIL', 'OENOTHERA BIENNIS'] | EVE PRIMROSE OIL |
| EVENING PRIMROSE OIL Synonyms: ['EVENING PRIMROSE OIL', 'OENOTHERA BIENNIS'] | OMEGA PRIMROSE OIL |
| EVENING PRIMROSE OIL Synonyms: ['EVENING PRIMROSE OIL', 'OENOTHERA BIENNIS'] | MOCCASION EVENING PRIMOSE |
| EVENING PRIMROSE OIL Synonyms: ['EVENING PRIMROSE OIL', 'OENOTHERA BIENNIS'] | DAILY KRILL OIL WEVENING PRIMROSE |
| EVENING PRIMROSE OIL Synonyms: ['EVENING PRIMROSE OIL', 'OENOTHERA BIENNIS'] | EVE PRIM OIL |
| EVENING PRIMROSE OIL Synonyms: ['EVENING PRIMROSE OIL', 'OENOTHERA BIENNIS'] | EVE PRIMROSE |
| EVENING PRIMROSE OIL Synonyms: ['EVENING PRIMROSE OIL', 'OENOTHERA BIENNIS'] | PRIMEROSE OIL |
| FENNEL Synonyms: ['FOENICULUM VULGARE', 'FENNEL'] | FENNEL TEA |
| FENUGREEK Synonyms: ['TRIGONELFA FOENUM', 'FENUGREEK'] | PHENOGREEK |
| FLAX SEED Synonyms: ['LINUM USITATISSIMUM', 'FLAX SEED'] | D B FLAX SEED OIL |
| FLAX SEED Synonyms: ['LINUM USITATISSIMUM', 'FLAX SEED'] | FAX SEED OIL |
| FLAX SEED Synonyms: ['LINUM USITATISSIMUM', 'FLAX SEED'] | FLEA SEEDS |
| GANODERMA LUCIDUM Synonyms: ['REISHI', 'GANODERMA LUCIDUM'] | GANODERMA TEA |
| GARCINIA GUMMI Synonyms: ['GARCINIA GUMMI', 'GARCINIA'] | GARCINIA CAMBOGIAALOE |
| GARCINIA GUMMI Synonyms: ['GARCINIA GUMMI', 'GARCINIA'] | GARCINIA FUCUS |
| GARCINIA GUMMI Synonyms: ['GARCINIA GUMMI', 'GARCINIA'] | GARCINIA SPP |
| GARCINIA GUMMI Synonyms: ['GARCINIA GUMMI', 'GARCINIA'] | GARCENIA CAMBOGIA |
| GARCINIA GUMMI Synonyms: ['GARCINIA GUMMI', 'GARCINIA'] | GARCINIA PLUS |
| GARCINIA Synonyms: ['GARCINIA GUMMI', 'GARCINIA'] | GARCINIA PLUS |
| GARCINIA Synonyms: ['GARCINIA GUMMI', 'GARCINIA'] | GARCINIA FUCUS |
| GARCINIA Synonyms: ['GARCINIA GUMMI', 'GARCINIA'] | CARCINIA |
| GARLIC Synonyms: ['GARLIC', 'ALLIUM SATIVUM'] | GARLIS |
| GARLIC Synonyms: ['GARLIC', 'ALLIUM SATIVUM'] | GARLIQUW |
| GINGER Synonyms: ['GINGER', 'ZINGIBER OFFICINALE'] | GINGEMBRE |
| GINKGO BILOBA Synonyms: ['GINKGO BILOBA', 'GINKGO'] | GINGKOMIN GINKGO BILOBA |
| GINKGO BILOBA Synonyms: ['GINKGO BILOBA', 'GINKGO'] | GINGKOBIL |
| GINKGO BILOBA Synonyms: ['GINKGO BILOBA', 'GINKGO'] | GINKOFAR GINKGO BILOBA |
| GINKGO BILOBA Synonyms: ['GINKGO BILOBA', 'GINKGO'] | SYMFONA GINKGO BILOBA |
| GINKGO BILOBA Synonyms: ['GINKGO BILOBA', 'GINKGO'] | GINKGO BILOBA UNKNOWN |
| GINKGO BILOBA Synonyms: ['GINKGO BILOBA', 'GINKGO'] | TANAKAN GINKGO BILOBA |
| GINKGO BILOBA Synonyms: ['GINKGO BILOBA', 'GINKGO'] | TEBONIN GINKGO BILOBA |
| GINKGO BILOBA Synonyms: ['GINKGO BILOBA', 'GINKGO'] | GINKGO GINKGO BILOBE |
| GINKGO BILOBA Synonyms: ['GINKGO BILOBA', 'GINKGO'] | GINKGO GINKGO BILBOA |
| GINKGO BILOBA Synonyms: ['GINKGO BILOBA', 'GINKGO'] | GINGO BILOBA EXTRACT |
| GINKGO BILOBA Synonyms: ['GINKGO BILOBA', 'GINKGO'] | CILOSTAZOLGINKGO BILOBA |
| GINKGO BILOBA Synonyms: ['GINKGO BILOBA', 'GINKGO'] | TAVONIN GINKGO BILOBA |
| GINKGO Synonyms: ['GINKGO BILOBA', 'GINKGO'] | GINKOKUP |
| GINKGO Synonyms: ['GINKGO BILOBA', 'GINKGO'] | GINKOFAR |
| GINKGO Synonyms: ['GINKGO BILOBA', 'GINKGO'] | GINKOBA M |
| GINKGO Synonyms: ['GINKGO BILOBA', 'GINKGO'] | GINKGOBEN |
| GINKGO Synonyms: ['GINKGO BILOBA', 'GINKGO'] | GINKGO MAX |
| GINKGO Synonyms: ['GINKGO BILOBA', 'GINKGO'] | GINKOGIN |
| GINKGO Synonyms: ['GINKGO BILOBA', 'GINKGO'] | GINKOPRIM |
| GINKGO Synonyms: ['GINKGO BILOBA', 'GINKGO'] | GINKOMED |
| GINKGO Synonyms: ['GINKGO BILOBA', 'GINKGO'] | GINKGO DEW |
| GINKGO Synonyms: ['GINKGO BILOBA', 'GINKGO'] | GINKGO CON |
| GINKGO Synonyms: ['GINKGO BILOBA', 'GINKGO'] | GINKOSTAR |
| GOJI BERRY Synonyms: ['LYCIUM BARBARUM', 'GOJI BERRY'] | GOJI BAYAS |
| GOJI BERRY Synonyms: ['LYCIUM BARBARUM', 'GOJI BERRY'] | GOJI BERRY SUPPLEMENT |
| GYMNEMA SYLVESTRE Synonyms: ['GYMNEMA SYLVESTRE', 'MIRACLEFRUIT'] | SYLVESTRE |
| HEDERA HELIX Synonyms: ['HEDERA HELIX', 'IVY LEAF'] | PROSPAN HEDERA HELIX |
| HEDERA HELIX Synonyms: ['HEDERA HELIX', 'IVY LEAF'] | PROSPAN HEDERA HELIX LEAF |
| HEDERA HELIX Synonyms: ['HEDERA HELIX', 'IVY LEAF'] | HEDERAE HELIX FLUID EXT |
| HORSECHESTNUT Synonyms: ['AESCULUS HIPPOCASTANUM', 'HORSECHESTNUT'] | HORSE CHEST FOR VC |
| HORSECHESTNUT Synonyms: ['AESCULUS HIPPOCASTANUM', 'HORSECHESTNUT'] | HORSECHESTNUT COMPLEX |
| HYPERICUM PERFORATUM Synonyms: ['HYPERICUM PERFORATUM', 'ST JOHNSWORT'] | PERFORATUM |
| HYPERICUM PERFORATUM Synonyms: ['HYPERICUM PERFORATUM', 'ST JOHNSWORT'] | HYPERICIN HERBAL TEA |
| IVY LEAF Synonyms: ['HEDERA HELIX', 'IVY LEAF'] | IVY EXTRACT |
| KRATOM Synonyms: ['MITRAGYNA SPECIOSA', 'KRATOM'] | OPMS KRATOM |
| KRATOM Synonyms: ['MITRAGYNA SPECIOSA', 'KRATOM'] | GREEN KRATOM |
| LEPIDIUM MEYENII Synonyms: ['LEPIDIUM MEYENII', 'MACA'] | MACA PERUANA LIPIDIUM MEYENII |
| LINUM USITATISSIMUM Synonyms: ['LINUM USITATISSIMUM', 'FLAX SEED'] | TRIPLE OMEGA LINUM USITATISSIUM OIL |
| MACA Synonyms: ['LEPIDIUM MEYENII', 'MACA'] | MACAS |
| MATRICARIA CHAMOMILLA Synonyms: ['CHAMOMILE', 'MATRICARIA CHAMOMILLA'] | MATRICARIA RECUTITA |
| MATRICARIA CHAMOMILLA Synonyms: ['CHAMOMILE', 'MATRICARIA CHAMOMILLA'] | VALERIANCHAMOMILE |
| MATRICARIA CHAMOMILLA Synonyms: ['CHAMOMILE', 'MATRICARIA CHAMOMILLA'] | SEDATOL MATRICARIA RECUTITA |
| MATRICARIA CHAMOMILLA Synonyms: ['CHAMOMILE', 'MATRICARIA CHAMOMILLA'] | MELATONINCHAMOMILE |
| MILK THISTLE Synonyms: ['MILK THISTLE', 'SILYBUM MARIANUM'] | LIQUID MILK THISTLE |
| MILK THISTLE Synonyms: ['MILK THISTLE', 'SILYBUM MARIANUM'] | SUPER MILK THISTL |
| MILK THISTLE Synonyms: ['MILK THISTLE', 'SILYBUM MARIANUM'] | MILK THISTLE MG CAP |
| MILK THISTLE Synonyms: ['MILK THISTLE', 'SILYBUM MARIANUM'] | SUPER MILK THISTLE X |
| MILK THISTLE Synonyms: ['MILK THISTLE', 'SILYBUM MARIANUM'] | MILK THISTLE PLUS |
| MILK THISTLE Synonyms: ['MILK THISTLE', 'SILYBUM MARIANUM'] | MIK THISTLE FRUIT |
| MILK THISTLE Synonyms: ['MILK THISTLE', 'SILYBUM MARIANUM'] | SUPER MILK THISTLE |
| MILK THISTLE Synonyms: ['MILK THISTLE', 'SILYBUM MARIANUM'] | MILK THISTLE CAP MG |
| MILK THISTLE Synonyms: ['MILK THISTLE', 'SILYBUM MARIANUM'] | CVS MILK THISLE |
| MILK THISTLE Synonyms: ['MILK THISTLE', 'SILYBUM MARIANUM'] | MILK THISTLE COMBO |
| MILK THISTLE Synonyms: ['MILK THISTLE', 'SILYBUM MARIANUM'] | PSYLIUM MILK THISTLE |
| MILK THISTLE Synonyms: ['MILK THISTLE', 'SILYBUM MARIANUM'] | MILK THRISTLE CON |
| MILK THISTLE Synonyms: ['MILK THISTLE', 'SILYBUM MARIANUM'] | MILKTHISTLE LIVER |
| MILK THISTLE Synonyms: ['MILK THISTLE', 'SILYBUM MARIANUM'] | MILK THISTLE XTRA |
| MILK THISTLE Synonyms: ['MILK THISTLE', 'SILYBUM MARIANUM'] | MILK THISTLE CAPS |
| MITRAGYNA SPECIOSA Synonyms: ['MITRAGYNA SPECIOSA', 'KRATOM'] | MITRAGYNA SPECIOSA KRATOM WVB CAPSULES |
| OLEA EUROPAEA Synonyms: ['OLIVE LEAF', 'OLEA EUROPAEA'] | OLIVE OIL OLEA EUROPAEA OIL |
| OLEA EUROPAEA Synonyms: ['OLIVE LEAF', 'OLEA EUROPAEA'] | TAE BULK OLEA EUROPAEA |
| OLEA EUROPAEA Synonyms: ['OLIVE LEAF', 'OLEA EUROPAEA'] | OLEA EUROPAEA GLUCOSATRIN |
| OLEA EUROPAEA Synonyms: ['OLIVE LEAF', 'OLEA EUROPAEA'] | OLEA EUROPAEA OIL SOYA OIL |
| OLEA EUROPAEA Synonyms: ['OLIVE LEAF', 'OLEA EUROPAEA'] | OLIVE OIL OLEA EUROPEA OIL |
| OPHIOCORDYCEPS SINENSIS Synonyms: ['CORDYCEPS', 'OPHIOCORDYCEPS SINENSIS'] | CORDYCEPS SINENSIS ADRENAL ESSENCE |
| OPHIOCORDYCEPS SINENSIS Synonyms: ['CORDYCEPS', 'OPHIOCORDYCEPS SINENSIS'] | CORDYCEPS MUSHROOMS |
| OPHIOCORDYCEPS SINENSIS Synonyms: ['CORDYCEPS', 'OPHIOCORDYCEPS SINENSIS'] | ORGANIC CORDYCEPS |
| OPHIOCORDYCEPS SINENSIS Synonyms: ['CORDYCEPS', 'OPHIOCORDYCEPS SINENSIS'] | CORDYCEPS POWDER |
| OPHIOCORDYCEPS SINENSIS Synonyms: ['CORDYCEPS', 'OPHIOCORDYCEPS SINENSIS'] | AGARICUS SUBRUFESCENS WCORDYCEPS SINENSISGA |
| OPHIOCORDYCEPS SINENSIS Synonyms: ['CORDYCEPS', 'OPHIOCORDYCEPS SINENSIS'] | CORDYCEPS ACTIV |
| OPHIOCORDYCEPS SINENSIS Synonyms: ['CORDYCEPS', 'OPHIOCORDYCEPS SINENSIS'] | CORDYCEPS CEPHALOSPORIUM |
| OREGANO Synonyms: ['ORIGANUM VULGARE', 'OREGANO'] | WILD OREGANO |
| PANAX GINSENG Synonyms: ['PANAX GINSENG'] | RED GINSENG |
| PANAX GINSENG Synonyms: ['PANAX GINSENG'] | KOREAN GINSING |
| PANAX GINSENG Synonyms: ['PANAX GINSENG'] | KOREAN GINSENG |
| PANAX GINSENG Synonyms: ['PANAX GINSENG'] | TRIPLE GINSENG |
| PIPER METHYSTICUM Synonyms: ['PIPER METHYSTICUM', 'KAVA'] | KAVA PIPER METHYSTICUM RHIZOME |
| PIPER METHYSTICUM Synonyms: ['PIPER METHYSTICUM', 'KAVA'] | KAVA KAVA PIPER METHYSTICUM ROOT |
| PIPER METHYSTICUM Synonyms: ['PIPER METHYSTICUM', 'KAVA'] | KAVA KAVA PIPER METHYSTICUM |
| PIPER METHYSTICUM Synonyms: ['PIPER METHYSTICUM', 'KAVA'] | PIPER METHYSTICUM ROOT |
| PRUNUS SEROTINA Synonyms: ['PRUNUS SEROTINA', 'BLACK CHERRY'] | WILD CHERRY PRUNUS SEROTINA |
| PRUNUS SEROTINA Synonyms: ['PRUNUS SEROTINA', 'BLACK CHERRY'] | BROCIN PRUNUS SEROTINA BARK |
| RED YEAST RICE Synonyms: ['ORYZA SATIVA', 'RED YEAST RICE'] | PYGEUM RED YEAST RICE |
| RED YEAST RICE Synonyms: ['ORYZA SATIVA', 'RED YEAST RICE'] | VO Q PLUS READ YEAST RICE |
| RED YEAST RICE Synonyms: ['ORYZA SATIVA', 'RED YEAST RICE'] | VIT D RED YEAST RICE |
| RED YEAST RICE Synonyms: ['ORYZA SATIVA', 'RED YEAST RICE'] | LDL RED YEAST |
| RED YEAST RICE Synonyms: ['ORYZA SATIVA', 'RED YEAST RICE'] | REDYEAST |
| SAMBUCUS NIGRA Synonyms: ['ELDERBERRY', 'SAMBUCUS NIGRA'] | ELDERBERRY SAMBUCUS NIGRA FRUIT |
| SAMBUCUS NIGRA Synonyms: ['ELDERBERRY', 'SAMBUCUS NIGRA'] | SAMBUCUS NIGRA HOMEOPATHICS |
| SCRUBPALMETTO Synonyms: ['SCRUBPALMETTO', 'SERENOA REPENS'] | PALMETTO MG |
| SCRUBPALMETTO Synonyms: ['SCRUBPALMETTO', 'SERENOA REPENS'] | HERBAL SAW PALMETTO |
| SCRUBPALMETTO Synonyms: ['SCRUBPALMETTO', 'SERENOA REPENS'] | PALMETTO PLUS |
| SCRUBPALMETTO Synonyms: ['SCRUBPALMETTO', 'SERENOA REPENS'] | PALMELTO |
| SENNA ALEXANDRINA Synonyms: ['SENNA ALEXANDRINA', 'SENNA'] | SENOKOTXTRA SENNA ALEXANDRINA |
| SENNA ALEXANDRINA Synonyms: ['SENNA ALEXANDRINA', 'SENNA'] | PIMURO SENNA ALEXANDRINA |
| SENNA ALEXANDRINA Synonyms: ['SENNA ALEXANDRINA', 'SENNA'] | SENOKOTSENNA ALEXANDRINA |
| SENNA ALEXANDRINA Synonyms: ['SENNA ALEXANDRINA', 'SENNA'] | CASSIA ALEXANDRINA |
| SENNA ALEXANDRINA Synonyms: ['SENNA ALEXANDRINA', 'SENNA'] | SENOKOT USA SENNA ALEXANDRINA |
| SENNA ALEXANDRINA Synonyms: ['SENNA ALEXANDRINA', 'SENNA'] | XPREP SENNA ALEXANDRINA LEAF |
| SENNA ALEXANDRINA Synonyms: ['SENNA ALEXANDRINA', 'SENNA'] | SENOKOT XTRA SENNA ALEXANDRINA |
| SENNA ALEXANDRINA Synonyms: ['SENNA ALEXANDRINA', 'SENNA'] | SENNA ALEXANDRINA SENOKOT |
| SENNA ALEXANDRINA Synonyms: ['SENNA ALEXANDRINA', 'SENNA'] | SENOKOT USASENNA ALEXANDRINA |
| SENNA ALEXANDRINA Synonyms: ['SENNA ALEXANDRINA', 'SENNA'] | SENOKOT SENNA ALEXANDRIA |
| SENNA ALEXANDRINA Synonyms: ['SENNA ALEXANDRINA', 'SENNA'] | SENOKOT SENNA ALEXANDRINA |
| SENNA ALEXANDRINA Synonyms: ['SENNA ALEXANDRINA', 'SENNA'] | SENNA ALEXANDRINA FRUIT |
| SENNA Synonyms: ['SENNA ALEXANDRINA', 'SENNA'] | SINNA |
| SENNA Synonyms: ['SENNA ALEXANDRINA', 'SENNA'] | SENNA C |
| SENNA Synonyms: ['SENNA ALEXANDRINA', 'SENNA'] | SENNAE |
| SENNA Synonyms: ['SENNA ALEXANDRINA', 'SENNA'] | SENNAL |
| SENNA Synonyms: ['SENNA ALEXANDRINA', 'SENNA'] | SENNAR |
| SENNA Synonyms: ['SENNA ALEXANDRINA', 'SENNA'] | SENNTAB |
| SERENOA REPENS Synonyms: ['SCRUBPALMETTO', 'SERENOA REPENS'] | PERMIXON SERENEA REPENS |
| SERENOA REPENS Synonyms: ['SCRUBPALMETTO', 'SERENOA REPENS'] | EVIPROSTAT SERENOA REPENS |
| SERENOA REPENS Synonyms: ['SCRUBPALMETTO', 'SERENOA REPENS'] | PROSTAMOL SERENOA REPENS |
| SILYBUM MARIANUM Synonyms: ['MILK THISTLE', 'SILYBUM MARIANUM'] | MLK THISTHLE SILYBUM MARIANUM |
| SILYBUM MARIANUM Synonyms: ['MILK THISTLE', 'SILYBUM MARIANUM'] | SILYBUM MARIANUM FRUIT |
| SILYBUM MARIANUM Synonyms: ['MILK THISTLE', 'SILYBUM MARIANUM'] | LEGALON SILYBUM MARIANUM |
| SILYBUM MARIANUM Synonyms: ['MILK THISTLE', 'SILYBUM MARIANUM'] | LAGOSA SILYBUM MARIANUM |
| SILYBUM MARIANUM Synonyms: ['MILK THISTLE', 'SILYBUM MARIANUM'] | SILY MARIN |
| SILYBUM MARIANUM Synonyms: ['MILK THISTLE', 'SILYBUM MARIANUM'] | LEGALONSILYBUM MARIANUM |
| SILYBUM MARIANUM Synonyms: ['MILK THISTLE', 'SILYBUM MARIANUM'] | SILLYMARIN |
| SILYBUM MARIANUM Synonyms: ['MILK THISTLE', 'SILYBUM MARIANUM'] | METHIONINE WSILYBUM MARIANUM |
| SILYBUM MARIANUM Synonyms: ['MILK THISTLE', 'SILYBUM MARIANUM'] | LIVERAID SILYBUM MARIANUM |
| SILYBUM MARIANUM Synonyms: ['MILK THISTLE', 'SILYBUM MARIANUM'] | FLAVOBION SILYBUM MARIANUM |
| ST JOHNSWORT Synonyms: ['HYPERICUM PERFORATUM', 'ST JOHNSWORT'] | ST JOHN WORT OTC |
| ST JOHNSWORT Synonyms: ['HYPERICUM PERFORATUM', 'ST JOHNSWORT'] | ST JOHNS WART MG |
| ST JOHNSWORT Synonyms: ['HYPERICUM PERFORATUM', 'ST JOHNSWORT'] | ST JOHNS WARTS |
| ST JOHNSWORT Synonyms: ['HYPERICUM PERFORATUM', 'ST JOHNSWORT'] | ST JOHNS WO |
| ST JOHNSWORT Synonyms: ['HYPERICUM PERFORATUM', 'ST JOHNSWORT'] | ST JOHNS WORT PLUS |
| STINGING NETTLE Synonyms: ['URTICA DIOICA', 'STINGING NETTLE'] | QUEROETINNETTLE |
| STINGING NETTLE Synonyms: ['URTICA DIOICA', 'STINGING NETTLE'] | QUERCETINNETTLE |
| STINGING NETTLE Synonyms: ['URTICA DIOICA', 'STINGING NETTLE'] | STINGING METTLE LEAF EXTRACT |
| TARAXACUM OFFICINALE Synonyms: ['LIONSTOOTH', 'TARAXACUM OFFICINALE'] | ORAL HYPOGLYCAEMIC AGENTS TARAXACUM OFFICINALE |
| TARAXACUM OFFICINALE Synonyms: ['LIONSTOOTH', 'TARAXACUM OFFICINALE'] | MILK THISTLE PLUS TARAXACUM OFFICINALE ROOT |
| TRITICUM AESTIVUM Synonyms: ['WHEAT GRASS', 'TRITICUM AESTIVUM'] | DAE BULK TRITICUM AESTIVUM |
| TRITICUM AESTIVUM Synonyms: ['WHEAT GRASS', 'TRITICUM AESTIVUM'] | FIBERFORM TRITICUM AESTIVUM |
| TURMERIC Synonyms: ['TURMERIC', 'CURCUMA JONGA'] | B TUMERIC |
| TURMERIC Synonyms: ['TURMERIC', 'CURCUMA JONGA'] | GCTUMERIC |
| TURMERIC Synonyms: ['TURMERIC', 'CURCUMA JONGA'] | TURMERIC ACID |
| TURMERIC Synonyms: ['TURMERIC', 'CURCUMA JONGA'] | TURMERIC ROOT |
| TURMERIC Synonyms: ['TURMERIC', 'CURCUMA JONGA'] | TURMERIC DS |
| TURMERIC Synonyms: ['TURMERIC', 'CURCUMA JONGA'] | TUMERIC TEA |
| TURMERIC Synonyms: ['TURMERIC', 'CURCUMA JONGA'] | TURMERICOMEGA |
| TURMERIC Synonyms: ['TURMERIC', 'CURCUMA JONGA'] | TURMERIC TEA |
| TURMERIC Synonyms: ['TURMERIC', 'CURCUMA JONGA'] | TURMERIC CBD |
| URTICA DIOICA Synonyms: ['URTICA DIOICA', 'STINGING NETTLE'] | DAE BULK URTICA DIOICA |
| VACCINIUM MACROCARPON Synonyms: ['VACCINIUM MACROCARPON', 'CRANBERRY'] | ASCORBIC ACID WVACCINIUM MACROCARPON |
| VACCINIUM MACROCARPON Synonyms: ['VACCINIUM MACROCARPON', 'CRANBERRY'] | ASCORBIC ACIDURTICA SPPVACCINIUM MACROCARPONZINC |
| VACCINIUM MACROCARPON Synonyms: ['VACCINIUM MACROCARPON', 'CRANBERRY'] | ASCORBIC ACID VACCINIUM MACROCARPON EXTRACT |
| VACCINIUM MACROCARPON Synonyms: ['VACCINIUM MACROCARPON', 'CRANBERRY'] | CRANMAX VACCINIUM MACROCAPRON |
| VACCINIUM MACROCARPON Synonyms: ['VACCINIUM MACROCARPON', 'CRANBERRY'] | URINAL VACCINIUM MACROCARPON |
| VACCINIUM MACROCARPON Synonyms: ['VACCINIUM MACROCARPON', 'CRANBERRY'] | ASCORBIC ACIDVACCINIUM MACROCARPON FRUIT |
| VACCINIUM MACROCARPON Synonyms: ['VACCINIUM MACROCARPON', 'CRANBERRY'] | ASCORBIC ACIDVACCINIUM MACROCARPON |
| VALERIANA OFFICINALIS Synonyms: ['VALERIAN', 'VALERIANA OFFICINALIS'] | BALDRIAN VALERIANA OFFICIALIS ROOT |
| VALERIANA OFFICINALIS Synonyms: ['VALERIAN', 'VALERIANA OFFICINALIS'] | VALERIAN ROOTVALERIANA OFFICINALIS ROOT |
| VALERIANA OFFICINALIS Synonyms: ['VALERIAN', 'VALERIANA OFFICINALIS'] | VALERIAN VALERIANA OFFICINALIS ROOT |
| VALERIANA OFFICINALIS Synonyms: ['VALERIAN', 'VALERIANA OFFICINALIS'] | VALVERDE VALERIANA OFFICINALIS EXTRACT |
| VALERIANA OFFICINALIS Synonyms: ['VALERIAN', 'VALERIANA OFFICINALIS'] | VALERIANA OFFICINALIS ROOT DRY EXTRACT |
| VALERIANA OFFICINALIS Synonyms: ['VALERIAN', 'VALERIANA OFFICINALIS'] | BALDRIAN VALERIANA OFFICINALIS ROOT |
| VALERIANA OFFICINALIS Synonyms: ['VALERIAN', 'VALERIANA OFFICINALIS'] | BALDRIAN VALERIANA OFFICINALIS |
| VALERIANA OFFICINALIS Synonyms: ['VALERIAN', 'VALERIANA OFFICINALIS'] | THIAMINEVALERIANA OFFICINALIS |
| VALERIANA OFFICINALIS Synonyms: ['VALERIAN', 'VALERIANA OFFICINALIS'] | NEUROL VALERIANA OFFICINALIS EXTRACT |
| WHEAT GRASS Synonyms: ['WHEAT GRASS', 'TRITICUM AESTIVUM'] | WHEATGRASS POWDER |
| WHEAT GRASS Synonyms: ['WHEAT GRASS', 'TRITICUM AESTIVUM'] | WHEAT GRASS JUICE |
| WHEAT GRASS Synonyms: ['WHEAT GRASS', 'TRITICUM AESTIVUM'] | WHEAT GRASS POWDER |
| WHEAT GRASS Synonyms: ['WHEAT GRASS', 'TRITICUM AESTIVUM'] | WHEATGRASS JUICE |
| WHEAT GRASS Synonyms: ['WHEAT GRASS', 'TRITICUM AESTIVUM'] | ORGANIC WHEAT GRASS |
| WOODLAND HAWTHORN Synonyms: ['WOODLAND HAWTHORN', 'CRATAEGUS LAEVIGATA'] | HAWTHORN MG |
| WOODLAND HAWTHORN Synonyms: ['WOODLAND HAWTHORN', 'CRATAEGUS LAEVIGATA'] | HAWTHORN |
| WOODLAND HAWTHORN Synonyms: ['WOODLAND HAWTHORN', 'CRATAEGUS LAEVIGATA'] | HAWTHORN EXT |
| WOODLAND HAWTHORN Synonyms: ['WOODLAND HAWTHORN', 'CRATAEGUS LAEVIGATA'] | HAWTHORN CAP |
| WOODLAND HAWTHORN Synonyms: ['WOODLAND HAWTHORN', 'CRATAEGUS LAEVIGATA'] | POWDERED HAWTHORNE BERRY |
| YOHIMBE Synonyms: ['YOHIMBE', 'PAUSINYSTALIA JOHIMBE'] | YOHIMBE BARK |
| ACAI Synonyms: ['EUTERPE OLERACEA', 'ACAI'] | ACAI |

## Natural Product Name Variations Identified in FAERS Reports by Both Approaches:

| **Query NP name** | **Identified drug string** |
| --- | --- |
| ACAI Synonyms: ['EUTERPE OLERACEA', 'ACAI'] | ACAI |
| ACAI Synonyms: ['EUTERPE OLERACEA', 'ACAI'] | ACAQI |
| AESCULUS HIPPOCASTANUM Synonyms: ['AESCULUS HIPPOCASTANUM', 'HORSECHESTNUT'] | AESCULUS HIPPOCASTANUM I INDIAN NUT |
| AESCULUS HIPPOCASTANUM Synonyms: ['AESCULUS HIPPOCASTANUM', 'HORSECHESTNUT'] | AESCULUS AESCULUS HIPPOCASTANUM |
| APPLE CIDER VINEGAR Synonyms: ['APPLE CIDER VINEGAR', 'MALUS DOMESTICA', 'MALUS PUMILA'] | CIGAR VINEGAR |
| BARLEY GRASS Synonyms: ['HORDEUM VULGARE', 'BARLEY GRASS'] | BARLEY GRASS PILLS |
| BARLEY GRASS Synonyms: ['HORDEUM VULGARE', 'BARLEY GRASS'] | BARLEY TABLETS |
| BARLEY GRASS Synonyms: ['HORDEUM VULGARE', 'BARLEY GRASS'] | BARLEY GRASS POWDER |
| BEET ROOT Synonyms: ['BEET ROOT', 'BETA VULGARIS'] | BEET ROOT EXTRACT |
| BLACK CHERRY Synonyms: ['PRUNUS SEROTINA', 'BLACK CHERRY'] | BLACK CHERRY PILL |
| BLACK CHERRY Synonyms: ['PRUNUS SEROTINA', 'BLACK CHERRY'] | BLACK CHERRY EXTRACT |
| BLACK CHERRY Synonyms: ['PRUNUS SEROTINA', 'BLACK CHERRY'] | BLACK CHERRY SYRUP |
| BLACK CHERRY Synonyms: ['PRUNUS SEROTINA', 'BLACK CHERRY'] | BLACK CHERRY CONC |
| BLACK CHERRY Synonyms: ['PRUNUS SEROTINA', 'BLACK CHERRY'] | BALCK CHERRY LIQ |
| BLACK COHOSH Synonyms: ['ACTAEA RACEMOSA', 'BLACK COHOSH'] | BLACK COHOSH HOT FLASH |
| BLACK COHOSH Synonyms: ['ACTAEA RACEMOSA', 'BLACK COHOSH'] | BLACK COHOSH |
| BLACK COHOSH Synonyms: ['ACTAEA RACEMOSA', 'BLACK COHOSH'] | BLACK COHOSH MG |
| BOSWELFIA SERRATA Synonyms: ['BOSWELFIA SERRATA', 'BOSWELLIA'] | BOSWELIA |
| BOSWELFIA SERRATA Synonyms: ['BOSWELFIA SERRATA', 'BOSWELLIA'] | BOSWELLI SERRATA DRY EXTRACT |
| BOSWELLIA Synonyms: ['BOSWELFIA SERRATA', 'BOSWELLIA'] | BOSWELLA |
| BOSWELLIA Synonyms: ['BOSWELFIA SERRATA', 'BOSWELLIA'] | BOSWILLIA |
| BOSWELLIA Synonyms: ['BOSWELFIA SERRATA', 'BOSWELLIA'] | BOSWELLIN |
| BOSWELLIA Synonyms: ['BOSWELFIA SERRATA', 'BOSWELLIA'] | BOSWELLIACURUMEN |
| BOSWELLIA Synonyms: ['BOSWELFIA SERRATA', 'BOSWELLIA'] | BOSWELLIACALCIUM |
| BOSWELLIA Synonyms: ['BOSWELFIA SERRATA', 'BOSWELLIA'] | BOSWELLIA ACID |
| BOSWELLIA Synonyms: ['BOSWELFIA SERRATA', 'BOSWELLIA'] | BOSWELIA |
| BOSWELLIA Synonyms: ['BOSWELFIA SERRATA', 'BOSWELLIA'] | BOSWEILLA |
| BOSWELLIA Synonyms: ['BOSWELFIA SERRATA', 'BOSWELLIA'] | BOSEWEILLA |
| CANNABIS SATIVA Synonyms: ['HEMP EXTRACT', 'CANNABIS SATIVA'] | CBD COMPLEX CANNABIS SATIVA |
| CANNABIS SATIVA Synonyms: ['HEMP EXTRACT', 'CANNABIS SATIVA'] | THC CANNABIS SATIVA |
| CANNABIS SATIVA Synonyms: ['HEMP EXTRACT', 'CANNABIS SATIVA'] | CANNABISCANNABIS SATIVA |
| CANNABIS SATIVA Synonyms: ['HEMP EXTRACT', 'CANNABIS SATIVA'] | THC CANNABIS STAIVA |
| CANNABIS SATIVA Synonyms: ['HEMP EXTRACT', 'CANNABIS SATIVA'] | CANNABIS CANNABIS CANNABIS SATIVA |
| CANNABIS SATIVA Synonyms: ['HEMP EXTRACT', 'CANNABIS SATIVA'] | MARIJUANA NOS CANNABIS SATIVA |
| CANNABIS SATIVA Synonyms: ['HEMP EXTRACT', 'CANNABIS SATIVA'] | CANNABIS CANNABIS SATIVA |
| CANNABIS SATIVA Synonyms: ['HEMP EXTRACT', 'CANNABIS SATIVA'] | CANNABIS SATIVA E SEMINIBUS |
| CANNABIS SATIVA Synonyms: ['HEMP EXTRACT', 'CANNABIS SATIVA'] | CANNABIS SATIVACANNABIS CANNABIS SATIVA |
| CANNABIS SATIVA Synonyms: ['HEMP EXTRACT', 'CANNABIS SATIVA'] | CANNABIS SATIVA CANNABIS SATIVA |
| CANNABIS SATIVA Synonyms: ['HEMP EXTRACT', 'CANNABIS SATIVA'] | CANNABIS CANNIBIS SATIVA |
| CANNABIS SATIVA Synonyms: ['HEMP EXTRACT', 'CANNABIS SATIVA'] | CANNABIS CANNABIS SATIVA CON |
| CATSCLAW Synonyms: ['UNCARIA TOMENTOSA', 'CATSCLAW'] | CATS CLAW |
| CHAMOMILE Synonyms: ['CHAMOMILE', 'MATRICARIA CHAMOMILLA'] | CHAMOMILE |
| CHAMOMILE Synonyms: ['CHAMOMILE', 'MATRICARIA CHAMOMILLA'] | CAMOMILIA |
| CHAMOMILE Synonyms: ['CHAMOMILE', 'MATRICARIA CHAMOMILLA'] | CAMOMILLA |
| CHLORELLA Synonyms: ['CHLORELLA VULGARIS', 'CHLORELLA'] | CHORELLA |
| CHLORELLA Synonyms: ['CHLORELLA VULGARIS', 'CHLORELLA'] | CHLORELLA |
| CHLORELLA Synonyms: ['CHLORELLA VULGARIS', 'CHLORELLA'] | COQCHLORELLA |
| CHLORELLA Synonyms: ['CHLORELLA VULGARIS', 'CHLORELLA'] | CHLORELA |
| CHLORELLA Synonyms: ['CHLORELLA VULGARIS', 'CHLORELLA'] | CHIORELLA |
| CHLORELLA Synonyms: ['CHLORELLA VULGARIS', 'CHLORELLA'] | CHLONELLA |
| CHLORELLA VULGARIS Synonyms: ['CHLORELLA VULGARIS', 'CHLORELLA'] | CHLORELLA CHLORELLA |
| CHLORELLA VULGARIS Synonyms: ['CHLORELLA VULGARIS', 'CHLORELLA'] | CHLORELLA |
| CINNAMOMUM CASSIA Synonyms: ['CINNAMOMUM VERUM', 'CINNAMOMUM CASSIA', 'CINNAMON'] | CINAMMIN |
| CINNAMOMUM CASSIA Synonyms: ['CINNAMOMUM VERUM', 'CINNAMOMUM CASSIA', 'CINNAMON'] | CINAMMON CAPSULE |
| CINNAMOMUM CASSIA Synonyms: ['CINNAMOMUM VERUM', 'CINNAMOMUM CASSIA', 'CINNAMON'] | CINNAMIN |
| CINNAMOMUM CASSIA Synonyms: ['CINNAMOMUM VERUM', 'CINNAMOMUM CASSIA', 'CINNAMON'] | CINNAMOMUM HOMACCORD |
| CINNAMOMUM VERUM Synonyms: ['CINNAMOMUM VERUM', 'CINNAMOMUM CASSIA', 'CINNAMON'] | CINNAMOMUM HOMACCORD |
| CINNAMOMUM VERUM Synonyms: ['CINNAMOMUM VERUM', 'CINNAMOMUM CASSIA', 'CINNAMON'] | CINAMMON CAPSULE |
| CINNAMON Synonyms: ['CINNAMOMUM VERUM', 'CINNAMOMUM CASSIA', 'CINNAMON'] | CINAMMIN |
| CINNAMON Synonyms: ['CINNAMOMUM VERUM', 'CINNAMOMUM CASSIA', 'CINNAMON'] | CINNAMIN |
| CINNAMON Synonyms: ['CINNAMOMUM VERUM', 'CINNAMOMUM CASSIA', 'CINNAMON'] | COQ WITH CINAMON |
| CORDYCEPS Synonyms: ['CORDYCEPS', 'OPHIOCORDYCEPS SINENSIS'] | CORDYCEPS AKTIV |
| CORDYCEPS Synonyms: ['CORDYCEPS', 'OPHIOCORDYCEPS SINENSIS'] | CORCYPS |
| CRANBERRY Synonyms: ['VACCINIUM MACROCARPON', 'CRANBERRY'] | CRANBERY CAPS |
| CRANBERRY Synonyms: ['VACCINIUM MACROCARPON', 'CRANBERRY'] | CRAN BERRY |
| CURCUMA JONGA Synonyms: ['TURMERIC', 'CURCUMA JONGA'] | CURCUMASORB |
| CURCUMA JONGA Synonyms: ['TURMERIC', 'CURCUMA JONGA'] | KURKUMA CURCUMA LONGA |
| CURCUMA JONGA Synonyms: ['TURMERIC', 'CURCUMA JONGA'] | CURCUMA LONG |
| CURCUMA JONGA Synonyms: ['TURMERIC', 'CURCUMA JONGA'] | TURMERIC CURCUMA LONGA |
| CURCUMA JONGA Synonyms: ['TURMERIC', 'CURCUMA JONGA'] | CURCUMA LONGA TUMERIC |
| ECHINACEA ANGUSTIFOLIA Synonyms: ['ECHINACEA', 'ECHINACEA ANGUSTIFOLIA'] | ECHINACEA ECHINACEA ANGUSTIFOLIA |
| ECHINACEA ANGUSTIFOLIA Synonyms: ['ECHINACEA', 'ECHINACEA ANGUSTIFOLIA'] | ECHILNACEA ECHINACEA ANGUSTIFOLIA |
| ECHINACEA ANGUSTIFOLIA Synonyms: ['ECHINACEA', 'ECHINACEA ANGUSTIFOLIA'] | ECHINACEA ECHINACEA ANGUSTIFOLIA ROOT |
| ECHINACEA ANGUSTIFOLIA Synonyms: ['ECHINACEA', 'ECHINACEA ANGUSTIFOLIA'] | ECHINACEA ANGUSTIFOLIA DROPS |
| ECHINACEA ANGUSTIFOLIA Synonyms: ['ECHINACEA', 'ECHINACEA ANGUSTIFOLIA'] | ECHINACEA PLUS |
| ECHINACEA PURPUREA Synonyms: ['ECHINACEA PURPUREA', 'ECHINACEAPURPLE CONEFLOWER'] | ECHINACEA WGOLDENSEAL |
| ECHINACEA PURPUREA Synonyms: ['ECHINACEA PURPUREA', 'ECHINACEAPURPLE CONEFLOWER'] | ECHINACEA GOLDENSEAL |
| ECHINACEA PURPUREA Synonyms: ['ECHINACEA PURPUREA', 'ECHINACEAPURPLE CONEFLOWER'] | ECHINACEA C COMPLETE |
| ECHINACEA PURPUREA Synonyms: ['ECHINACEA PURPUREA', 'ECHINACEAPURPLE CONEFLOWER'] | ECHINACEA PLUS |
| ECHINACEA PURPUREA Synonyms: ['ECHINACEA PURPUREA', 'ECHINACEAPURPLE CONEFLOWER'] | ECHINACEA ECHINACEA PURPEA |
| ECHINACEA PURPUREA Synonyms: ['ECHINACEA PURPUREA', 'ECHINACEAPURPLE CONEFLOWER'] | ECHINACEAPLUS |
| ECHINACEA PURPUREA Synonyms: ['ECHINACEA PURPUREA', 'ECHINACEAPURPLE CONEFLOWER'] | ECHINACEA ECHINACEA PURPUREA HERB |
| ECHINACEA PURPUREA Synonyms: ['ECHINACEA PURPUREA', 'ECHINACEAPURPLE CONEFLOWER'] | ECHINACEAGOLDEN SEAL |
| ECHINACEA Synonyms: ['ECHINACEA', 'ECHINACEA ANGUSTIFOLIA'] | ZINCECHINACEA |
| ECHINACEA Synonyms: ['ECHINACEA', 'ECHINACEA ANGUSTIFOLIA'] | ECHINACEA ECHINACEA PURPUREA HERB |
| ECHINACEA Synonyms: ['ECHINACEA', 'ECHINACEA ANGUSTIFOLIA'] | ECANACIA |
| ECHINACEA Synonyms: ['ECHINACEA', 'ECHINACEA ANGUSTIFOLIA'] | ECHINACEA |
| ECHINACEA Synonyms: ['ECHINACEA', 'ECHINACEA ANGUSTIFOLIA'] | ECHINACEA ECHINACHEA PURPUREA ECHINACHEA PURPUREA |
| ECHINACEA Synonyms: ['ECHINACEA', 'ECHINACEA ANGUSTIFOLIA'] | ECCHINICAE |
| ECHINACEAPURPLE CONEFLOWER Synonyms: ['ECHINACEA PURPUREA', 'ECHINACEAPURPLE CONEFLOWER'] | ECHINACEAPLUS |
| ECHINACEAPURPLE CONEFLOWER Synonyms: ['ECHINACEA PURPUREA', 'ECHINACEAPURPLE CONEFLOWER'] | ECHINACEA PLUS |
| ECHINACEAPURPLE CONEFLOWER Synonyms: ['ECHINACEA PURPUREA', 'ECHINACEAPURPLE CONEFLOWER'] | ECHINACEA GOLDEN SEAL |
| ECHINACEAPURPLE CONEFLOWER Synonyms: ['ECHINACEA PURPUREA', 'ECHINACEAPURPLE CONEFLOWER'] | ECHINACEA ECHINACHEA PURPUREA ECHINACHEA PURPUREA |
| ECHINACEAPURPLE CONEFLOWER Synonyms: ['ECHINACEA PURPUREA', 'ECHINACEAPURPLE CONEFLOWER'] | ECHINACEA C COMPLETE |
| ECHINACEAPURPLE CONEFLOWER Synonyms: ['ECHINACEA PURPUREA', 'ECHINACEAPURPLE CONEFLOWER'] | ECHINACEA ECHINACEA PURPUREA HERB |
| ECHINACEAPURPLE CONEFLOWER Synonyms: ['ECHINACEA PURPUREA', 'ECHINACEAPURPLE CONEFLOWER'] | ECHINACEA ECHINACEA PURPURA ECHINACEA PURPURA |
| ECHINACEAPURPLE CONEFLOWER Synonyms: ['ECHINACEA PURPUREA', 'ECHINACEAPURPLE CONEFLOWER'] | ECHINACEA ECHINACEA PURPEA |
| ELDERBERRY Synonyms: ['ELDERBERRY', 'SAMBUCUS NIGRA'] | ELDERBERRY TINCTURE |
| ELDERBERRY Synonyms: ['ELDERBERRY', 'SAMBUCUS NIGRA'] | ELDERBERRY |
| ELDERBERRY Synonyms: ['ELDERBERRY', 'SAMBUCUS NIGRA'] | EDELBERRY ELDERBERRY |
| EUTERPE OLERACEA Synonyms: ['EUTERPE OLERACEA', 'ACAI'] | EUTERPE OLERACEA |
| EVENING PRIMROSE OIL Synonyms: ['EVENING PRIMROSE OIL', 'OENOTHERA BIENNIS'] | VEGEN PRIMROSE OIL |
| EVENING PRIMROSE OIL Synonyms: ['EVENING PRIMROSE OIL', 'OENOTHERA BIENNIS'] | EVENINGPRIMOSE EVENING PRIMOSE OIL |
| FENNEL Synonyms: ['FOENICULUM VULGARE', 'FENNEL'] | FENNEL |
| FENUGREEK Synonyms: ['TRIGONELFA FOENUM', 'FENUGREEK'] | FERNAGREK |
| FEVERFEW Synonyms: ['TANACETUM PARTHENIUM', 'FEVERFEW'] | FEVERFEW |
| FLAX SEED Synonyms: ['LINUM USITATISSIMUM', 'FLAX SEED'] | FLACK SEED OIL |
| FLAX SEED Synonyms: ['LINUM USITATISSIMUM', 'FLAX SEED'] | FLAK SEED OIL |
| FLAX SEED Synonyms: ['LINUM USITATISSIMUM', 'FLAX SEED'] | FLAXSEED OIL |
| FLAX SEED Synonyms: ['LINUM USITATISSIMUM', 'FLAX SEED'] | FLEX SEED OIL |
| FOENICULUM VULGARE Synonyms: ['FOENICULUM VULGARE', 'FENNEL'] | FENNEL FOENICULUM VULGARE SEED |
| GANODERMA LUCIDUM Synonyms: ['REISHI', 'GANODERMA LUCIDUM'] | REISHI GANODERMA LUCIDUM |
| GARCINIA GUMMI Synonyms: ['GARCINIA GUMMI', 'GARCINIA'] | GARCINIA GUMMIGUTTA |
| GARCINIA GUMMI Synonyms: ['GARCINIA GUMMI', 'GARCINIA'] | GARCINIA GAMBOGIA |
| GARCINIA GUMMI Synonyms: ['GARCINIA GUMMI', 'GARCINIA'] | GARCINIA CAMBOGIA GARCINIA GUMMIGUTTA FRUIT |
| GARCINIA GUMMI Synonyms: ['GARCINIA GUMMI', 'GARCINIA'] | GARCINIA CAMBODIA |
| GARCINIA GUMMI Synonyms: ['GARCINIA GUMMI', 'GARCINIA'] | GARCINIA CAMBOGIA |
| GARCINIA GUMMI Synonyms: ['GARCINIA GUMMI', 'GARCINIA'] | GARCINIA CAMBROGIA |
| GARCINIA GUMMI Synonyms: ['GARCINIA GUMMI', 'GARCINIA'] | GARCINIA |
| GARCINIA GUMMI Synonyms: ['GARCINIA GUMMI', 'GARCINIA'] | GARCINA CAMBOGIA |
| GARCINIA GUMMI Synonyms: ['GARCINIA GUMMI', 'GARCINIA'] | GARCINIA CAM |
| GARCINIA GUMMI Synonyms: ['GARCINIA GUMMI', 'GARCINIA'] | GARCINIA GUMMIGUTTA EXTRACT |
| GARCINIA Synonyms: ['GARCINIA GUMMI', 'GARCINIA'] | GARCINIA CAM |
| GARCINIA Synonyms: ['GARCINIA GUMMI', 'GARCINIA'] | GARCINIA |
| GARCINIA Synonyms: ['GARCINIA GUMMI', 'GARCINIA'] | GARCINIA SPP |
| GINKGO BILOBA Synonyms: ['GINKGO BILOBA', 'GINKGO'] | GINGKO GINKGO BILOBA |
| GINKGO BILOBA Synonyms: ['GINKGO BILOBA', 'GINKGO'] | GINKYO GINKGO BILOBA |
| GINKGO BILOBA Synonyms: ['GINKGO BILOBA', 'GINKGO'] | GINKGO BILOBA |
| GINKGO BILOBA Synonyms: ['GINKGO BILOBA', 'GINKGO'] | GINKGO GINKGO BILOBA |
| GINKGO BILOBA Synonyms: ['GINKGO BILOBA', 'GINKGO'] | GINKOBA GINKGO BILOBA |
| GINKGO BILOBA Synonyms: ['GINKGO BILOBA', 'GINKGO'] | GINKO BILBOA BILOBA |
| GINKGO BILOBA Synonyms: ['GINKGO BILOBA', 'GINKGO'] | GINKO GINKGO BILOBA |
| GINKGO BILOBA Synonyms: ['GINKGO BILOBA', 'GINKGO'] | GINGKO BILOBA GINKGO BILOBA |
| GINKGO BILOBA Synonyms: ['GINKGO BILOBA', 'GINKGO'] | GINKGOGINKGO BILOBA |
| GINKGO BILOBA Synonyms: ['GINKGO BILOBA', 'GINKGO'] | GINGKO BILOBA GINGKO BILOBA |
| GINKGO Synonyms: ['GINKGO BILOBA', 'GINKGO'] | GINGKO BILOBA GINKGO BILOBA |
| GINKGO Synonyms: ['GINKGO BILOBA', 'GINKGO'] | GINGKO BILOBA GINGKO BILOBA |
| GINKGO Synonyms: ['GINKGO BILOBA', 'GINKGO'] | GINGKOBA |
| GINKGO Synonyms: ['GINKGO BILOBA', 'GINKGO'] | GINKGO |
| GINKGO Synonyms: ['GINKGO BILOBA', 'GINKGO'] | GINKOBA |
| GINKGO Synonyms: ['GINKGO BILOBA', 'GINKGO'] | GINKOR |
| GINKGO Synonyms: ['GINKGO BILOBA', 'GINKGO'] | GINKO |
| GOJI BERRY Synonyms: ['LYCIUM BARBARUM', 'GOJI BERRY'] | GOJI BERRIES |
| GOJI BERRY Synonyms: ['LYCIUM BARBARUM', 'GOJI BERRY'] | GOGI BERRIES |
| GYMNEMA SYLVESTRE Synonyms: ['GYMNEMA SYLVESTRE', 'MIRACLEFRUIT'] | GYMMEMA SYLVESTRE LEAF ASTTRACT |
| HARPAGOPHYTUM PROCUMBENS Synonyms: ['HARPAGOPHYTUM PROCUMBENS', 'WOOD SPIDER'] | DEVILS CLAW HARPAGOPHYTUM PROCUMBENS |
| HEDERA HELIX Synonyms: ['HEDERA HELIX', 'IVY LEAF'] | HEDERA HELIX HEDERA HELIX |
| HEDERA HELIX Synonyms: ['HEDERA HELIX', 'IVY LEAF'] | IVY HEDERA HELIX LEAF |
| HEDERA HELIX Synonyms: ['HEDERA HELIX', 'IVY LEAF'] | IVY HEDERA HELIX DRY LEAF |
| HEMP EXTRACT Synonyms: ['HEMP EXTRACT', 'CANNABIS SATIVA'] | HEMP HEART |
| HEMP EXTRACT Synonyms: ['HEMP EXTRACT', 'CANNABIS SATIVA'] | CANNABIS CANNABIS CANNABIS SATIVA |
| HEMP EXTRACT Synonyms: ['HEMP EXTRACT', 'CANNABIS SATIVA'] | CANNABIS SATIVACANNABIS CANNABIS SATIVA |
| HEMP EXTRACT Synonyms: ['HEMP EXTRACT', 'CANNABIS SATIVA'] | CANNABIS SATIVA CANNABIS SATIVA |
| HORSECHESTNUT Synonyms: ['AESCULUS HIPPOCASTANUM', 'HORSECHESTNUT'] | HORSECHESTNUT EXTRACT |
| HORSECHESTNUT Synonyms: ['AESCULUS HIPPOCASTANUM', 'HORSECHESTNUT'] | AESCULUS AESCULUS HIPPOCASTANUM |
| HYPERICUM PERFORATUM Synonyms: ['HYPERICUM PERFORATUM', 'ST JOHNSWORT'] | ST JOHNS WORT HYPERICUM PERFORMATUM |
| HYPERICUM PERFORATUM Synonyms: ['HYPERICUM PERFORATUM', 'ST JOHNSWORT'] | HYPERICUM PERFORAMTUM ST JOHNS WORT TABLETS |
| IVY LEAF Synonyms: ['HEDERA HELIX', 'IVY LEAF'] | IVY HEDERA HELIX LEAF |
| KAVA Synonyms: ['PIPER METHYSTICUM', 'KAVA'] | KAVA |
| KRATOM Synonyms: ['MITRAGYNA SPECIOSA', 'KRATOM'] | KRATOM MG |
| LYCIUM BARBARUM Synonyms: ['LYCIUM BARBARUM', 'GOJI BERRY'] | LYCIUM BARBARUM L |
| LYCIUM BARBARUM Synonyms: ['LYCIUM BARBARUM', 'GOJI BERRY'] | LYCIUM BARBARUM HERBALS |
| MATRICARIA CHAMOMILLA Synonyms: ['CHAMOMILE', 'MATRICARIA CHAMOMILLA'] | CAMOMILLA |
| MILK THISTLE Synonyms: ['MILK THISTLE', 'SILYBUM MARIANUM'] | SILYBUM MARIANUM SYLBIUM MARIANUM |
| MILK THISTLE Synonyms: ['MILK THISTLE', 'SILYBUM MARIANUM'] | MILK THISTLE |
| MILK THISTLE Synonyms: ['MILK THISTLE', 'SILYBUM MARIANUM'] | MILK THISTLE CON |
| MILK THISTLE Synonyms: ['MILK THISTLE', 'SILYBUM MARIANUM'] | MILK THISTLE TABLET |
| MILK THISTLE Synonyms: ['MILK THISTLE', 'SILYBUM MARIANUM'] | MILK THISTLE MG |
| MILK THISTLE Synonyms: ['MILK THISTLE', 'SILYBUM MARIANUM'] | MILK THISTLE MILK THISTLE |
| MILK THISTLE Synonyms: ['MILK THISTLE', 'SILYBUM MARIANUM'] | MILK THISTLE OTC |
| MILK THISTLE Synonyms: ['MILK THISTLE', 'SILYBUM MARIANUM'] | SILYBUM MARIANUM SILYBUM MARIANUM |
| MITRAGYNA SPECIOSA Synonyms: ['MITRAGYNA SPECIOSA', 'KRATOM'] | KATROM MITRAGYNA SPECIOSA |
| MORINGA OLEIFERA Synonyms: ['MORINGA', 'MORINGA OLEIFERA'] | MORINGA MORINGA OLEIFERA |
| MORINGA Synonyms: ['MORINGA', 'MORINGA OLEIFERA'] | MORINGA |
| NIGELLA SATIVA Synonyms: ['BLACK CUMIN', 'NIGELLA SATIVA'] | BLACK SEED OIL NIGELLA SATIVA |
| NIGELLA SATIVA Synonyms: ['BLACK CUMIN', 'NIGELLA SATIVA'] | BLACK SEEDS NIGELLA SATIVA |
| OLEA EUROPAEA Synonyms: ['OLIVE LEAF', 'OLEA EUROPAEA'] | OLEA EUROPAEA LEAF |
| OLEA EUROPAEA Synonyms: ['OLIVE LEAF', 'OLEA EUROPAEA'] | OLEA EUROPAE |
| OLEA EUROPAEA Synonyms: ['OLIVE LEAF', 'OLEA EUROPAEA'] | OLIVE OIL OLEA EUROPEA |
| OLEA EUROPAEA Synonyms: ['OLIVE LEAF', 'OLEA EUROPAEA'] | OLIVE LEAF OLEA EUROPAEA LEAF |
| OLIVE LEAF Synonyms: ['OLIVE LEAF', 'OLEA EUROPAEA'] | OLIVE LEAF COMPLEX |
| OLIVE LEAF Synonyms: ['OLIVE LEAF', 'OLEA EUROPAEA'] | OLIVE LEAF EXTRACT II |
| OLIVE LEAF Synonyms: ['OLIVE LEAF', 'OLEA EUROPAEA'] | OLIVE LEAF |
| OPHIOCORDYCEPS SINENSIS Synonyms: ['CORDYCEPS', 'OPHIOCORDYCEPS SINENSIS'] | CORDYCEPS AKTIV |
| OPHIOCORDYCEPS SINENSIS Synonyms: ['CORDYCEPS', 'OPHIOCORDYCEPS SINENSIS'] | OPHIOCORDYCEPS SINENSIS |
| PAULLINIA CUPANA Synonyms: ['GUARANA', 'PAULLINIA CUPANA'] | PAULINA CUPANA PAULINA CUPANA |
| PRUNUS SEROTINA Synonyms: ['PRUNUS SEROTINA', 'BLACK CHERRY'] | PRUNUS SEROTINA BARK |
| RED YEAST RICE Synonyms: ['ORYZA SATIVA', 'RED YEAST RICE'] | RED YEAST CAP MG |
| RED YEAST RICE Synonyms: ['ORYZA SATIVA', 'RED YEAST RICE'] | RED YEAST CAB |
| RED YEAST RICE Synonyms: ['ORYZA SATIVA', 'RED YEAST RICE'] | RED YEAST CAP |
| RED YEAST RICE Synonyms: ['ORYZA SATIVA', 'RED YEAST RICE'] | RED YEAST CAPS |
| RED YEAST RICE Synonyms: ['ORYZA SATIVA', 'RED YEAST RICE'] | RED YEAST EXTRACT |
| RED YEAST RICE Synonyms: ['ORYZA SATIVA', 'RED YEAST RICE'] | RED YEAST RIC |
| RED YEAST RICE Synonyms: ['ORYZA SATIVA', 'RED YEAST RICE'] | RED YEAST RISE |
| RED YEAST RICE Synonyms: ['ORYZA SATIVA', 'RED YEAST RICE'] | RED YEAST RYE |
| RED YEAST RICE Synonyms: ['ORYZA SATIVA', 'RED YEAST RICE'] | RED YEAST CAPSULES |
| SAMBUCUS NIGRA Synonyms: ['ELDERBERRY', 'SAMBUCUS NIGRA'] | SAMBUCUS IMMUNE |
| SAMBUCUS NIGRA Synonyms: ['ELDERBERRY', 'SAMBUCUS NIGRA'] | ELDERBERRY SAMBUCUS NIGRA |
| SAMBUCUS NIGRA Synonyms: ['ELDERBERRY', 'SAMBUCUS NIGRA'] | EDERBERRY SAMBUCUS NIGRA |
| SAMBUCUS NIGRA Synonyms: ['ELDERBERRY', 'SAMBUCUS NIGRA'] | SAMBUCCUS ELDERBERRY |
| SAMBUCUS NIGRA Synonyms: ['ELDERBERRY', 'SAMBUCUS NIGRA'] | SAMBUCUS ELDERBERRY |
| SAMBUCUS NIGRA Synonyms: ['ELDERBERRY', 'SAMBUCUS NIGRA'] | ELDERBERRYSAMBUCUS NIGRA |
| SCRUBPALMETTO Synonyms: ['SCRUBPALMETTO', 'SERENOA REPENS'] | OTC SAWPALMETTO |
| SCRUBPALMETTO Synonyms: ['SCRUBPALMETTO', 'SERENOA REPENS'] | PLAMETTO |
| SENNA ALEXANDRINA Synonyms: ['SENNA ALEXANDRINA', 'SENNA'] | SENNASENNA ALEXANDRINA |
| SENNA ALEXANDRINA Synonyms: ['SENNA ALEXANDRINA', 'SENNA'] | EXLAX SENNA ALEXANDRINA |
| SENNA ALEXANDRINA Synonyms: ['SENNA ALEXANDRINA', 'SENNA'] | EXLAX SENNA SENNA ALEXANDRINA |
| SENNA ALEXANDRINA Synonyms: ['SENNA ALEXANDRINA', 'SENNA'] | SENNAUSASENNA ALEXANDRIA |
| SENNA ALEXANDRINA Synonyms: ['SENNA ALEXANDRINA', 'SENNA'] | SENNA ALEXANDRINA |
| SENNA ALEXANDRINA Synonyms: ['SENNA ALEXANDRINA', 'SENNA'] | SENNA SENNA ALEXANDRINA |
| SENNA ALEXANDRINA Synonyms: ['SENNA ALEXANDRINA', 'SENNA'] | SENNA SENNA ALEXANDRIA |
| SENNA ALEXANDRINA Synonyms: ['SENNA ALEXANDRINA', 'SENNA'] | SENNAGEN SENNA ALEXANDRINA |
| SENNA Synonyms: ['SENNA ALEXANDRINA', 'SENNA'] | SIENNA |
| SENNA Synonyms: ['SENNA ALEXANDRINA', 'SENNA'] | ASENNA |
| SENNA Synonyms: ['SENNA ALEXANDRINA', 'SENNA'] | SENNAS |
| SENNA Synonyms: ['SENNA ALEXANDRINA', 'SENNA'] | SENNAF |
| SENNA Synonyms: ['SENNA ALEXANDRINA', 'SENNA'] | SENNAC |
| SENNA Synonyms: ['SENNA ALEXANDRINA', 'SENNA'] | SENNAX |
| SENNA Synonyms: ['SENNA ALEXANDRINA', 'SENNA'] | SENNA |
| SENNA Synonyms: ['SENNA ALEXANDRINA', 'SENNA'] | SENA |
| SILYBUM MARIANUM Synonyms: ['MILK THISTLE', 'SILYBUM MARIANUM'] | MILK THRISTLE SILYBUM MARIANUM |
| SILYBUM MARIANUM Synonyms: ['MILK THISTLE', 'SILYBUM MARIANUM'] | MILK THISTLE MILK THISTLE |
| SILYBUM MARIANUM Synonyms: ['MILK THISTLE', 'SILYBUM MARIANUM'] | LIVER SILYBUM MARIANUM |
| SILYBUM MARIANUM Synonyms: ['MILK THISTLE', 'SILYBUM MARIANUM'] | MILK TRISTLE SILYBUM MARIANUM |
| SILYBUM MARIANUM Synonyms: ['MILK THISTLE', 'SILYBUM MARIANUM'] | MILK THISTLE SILYBUM MARIANUM |
| SILYBUM MARIANUM Synonyms: ['MILK THISTLE', 'SILYBUM MARIANUM'] | SILYBUM MARIANUM NGX |
| SILYBUM MARIANUM Synonyms: ['MILK THISTLE', 'SILYBUM MARIANUM'] | SILIBUM MARIANUM |
| SILYBUM MARIANUM Synonyms: ['MILK THISTLE', 'SILYBUM MARIANUM'] | SILYBUM MARIANUM SYLBIUM MARIANUM |
| SILYBUM MARIANUM Synonyms: ['MILK THISTLE', 'SILYBUM MARIANUM'] | SILYMARIN MARIANUM |
| SILYBUM MARIANUM Synonyms: ['MILK THISTLE', 'SILYBUM MARIANUM'] | SILYMARINUM |
| SILYBUM MARIANUM Synonyms: ['MILK THISTLE', 'SILYBUM MARIANUM'] | SILYMARIN SILYBUM MARIANUM |
| ST JOHNSWORT Synonyms: ['HYPERICUM PERFORATUM', 'ST JOHNSWORT'] | ST JOHNS WART |
| ST JOHNSWORT Synonyms: ['HYPERICUM PERFORATUM', 'ST JOHNSWORT'] | ST JOHN WORT |
| ST JOHNSWORT Synonyms: ['HYPERICUM PERFORATUM', 'ST JOHNSWORT'] | ST JHONS WORT |
| ST JOHNSWORT Synonyms: ['HYPERICUM PERFORATUM', 'ST JOHNSWORT'] | ST JOHNS WORT |
| ST JOHNSWORT Synonyms: ['HYPERICUM PERFORATUM', 'ST JOHNSWORT'] | JOHNS WORT |
| ST JOHNSWORT Synonyms: ['HYPERICUM PERFORATUM', 'ST JOHNSWORT'] | SAINT JOHNS WORT |
| ST JOHNSWORT Synonyms: ['HYPERICUM PERFORATUM', 'ST JOHNSWORT'] | NO ST JOHNS WORT |
| ST JOHNSWORT Synonyms: ['HYPERICUM PERFORATUM', 'ST JOHNSWORT'] | EQL ST JOHNS WORT |
| ST JOHNSWORT Synonyms: ['HYPERICUM PERFORATUM', 'ST JOHNSWORT'] | ST JOHNSWORT |
| ST JOHNSWORT Synonyms: ['HYPERICUM PERFORATUM', 'ST JOHNSWORT'] | SAINTJOHNS WORT |
| ST JOHNSWORT Synonyms: ['HYPERICUM PERFORATUM', 'ST JOHNSWORT'] | ST JOHNS WRT CAP |
| ST JOHNSWORT Synonyms: ['HYPERICUM PERFORATUM', 'ST JOHNSWORT'] | ST JOHNS WORTH |
| ST JOHNSWORT Synonyms: ['HYPERICUM PERFORATUM', 'ST JOHNSWORT'] | ST JOHNS WORT OIL |
| ST JOHNSWORT Synonyms: ['HYPERICUM PERFORATUM', 'ST JOHNSWORT'] | ST JOHNS WORT MG |
| ST JOHNSWORT Synonyms: ['HYPERICUM PERFORATUM', 'ST JOHNSWORT'] | STJOHNS WORT |
| STEVIA REBAUDIANA Synonyms: ['STEVIA REBAUDIANA', 'STEVIA'] | STEVIA STEVIA REBAUDIANA |
| STEVIA Synonyms: ['STEVIA REBAUDIANA', 'STEVIA'] | ATEVIA |
| STEVIA Synonyms: ['STEVIA REBAUDIANA', 'STEVIA'] | ESTAVIA |
| STEVIA Synonyms: ['STEVIA REBAUDIANA', 'STEVIA'] | STEVA |
| TARAXACUM OFFICINALE Synonyms: ['LIONSTOOTH', 'TARAXACUM OFFICINALE'] | TARAXCUM OFFICINALE TARAXACUM OFFICINALE |
| TURMERIC Synonyms: ['TURMERIC', 'CURCUMA JONGA'] | TURMERIC MAX |
| TURMERIC Synonyms: ['TURMERIC', 'CURCUMA JONGA'] | TURMERIC |
| TURMERIC Synonyms: ['TURMERIC', 'CURCUMA JONGA'] | RA TURMERIC |
| TURMERIC Synonyms: ['TURMERIC', 'CURCUMA JONGA'] | TUMERIC K |
| TURMERIC Synonyms: ['TURMERIC', 'CURCUMA JONGA'] | TUMERICCOQ |
| VACCINIUM MACROCARPON Synonyms: ['VACCINIUM MACROCARPON', 'CRANBERRY'] | CRANBERY CAPSULE VACCINIUM MACROCARPON |
| VACCINIUM MACROCARPON Synonyms: ['VACCINIUM MACROCARPON', 'CRANBERRY'] | VACCINIUM MACROCARPON VACCINIUM MACROCARPON |
| VALERIAN Synonyms: ['VALERIAN', 'VALERIANA OFFICINALIS'] | VALERIANA |
| VALERIAN Synonyms: ['VALERIAN', 'VALERIANA OFFICINALIS'] | VALERIAN |
| VALERIAN Synonyms: ['VALERIAN', 'VALERIANA OFFICINALIS'] | VALERIAAN |
| VALERIANA OFFICINALIS Synonyms: ['VALERIAN', 'VALERIANA OFFICINALIS'] | VALERIAN VALERIANA OFFICINALIS |
| VALERIANA OFFICINALIS Synonyms: ['VALERIAN', 'VALERIANA OFFICINALIS'] | VALERIANA OFFICINALIS TABLETS |
| VALERIANA OFFICINALIS Synonyms: ['VALERIAN', 'VALERIANA OFFICINALIS'] | VALERIANA OFFIMELISSA OFFI |
| VALERIANA OFFICINALIS Synonyms: ['VALERIAN', 'VALERIANA OFFICINALIS'] | VALERIN VALERIANA OFFICINALIS |
| VALERIANA OFFICINALIS Synonyms: ['VALERIAN', 'VALERIANA OFFICINALIS'] | VALERIAN ROOT VALERIANA OFFICINALIS |
| VALERIANA OFFICINALIS Synonyms: ['VALERIAN', 'VALERIANA OFFICINALIS'] | VALERIANVALERIANA OFFICINALIS |
| WHEAT GRASS Synonyms: ['WHEAT GRASS', 'TRITICUM AESTIVUM'] | WHEAT GRASS DRINK |
| WHEAT GRASS Synonyms: ['WHEAT GRASS', 'TRITICUM AESTIVUM'] | WEST WHEAT GRASS |
| ZINGIBER OFFICINALE Synonyms: ['GINGER', 'ZINGIBER OFFICINALE'] | GINGER ZINGIBER OFFICINALE ROOT |
| ZINGIBER OFFICINALE Synonyms: ['GINGER', 'ZINGIBER OFFICINALE'] | ZINTONA ZINGIBER OFFICINALE |
